# Elucidating the impact of point defects on the structural, electronic, and mechanical behaviour of chromium nitride

**DOI:** 10.1039/d5cp02904j

**Published:** 2025-10-07

**Authors:** Barsha Bhattacharjee, Emilia Olsson

**Affiliations:** a Institute of Theoretical Physics, University of Amsterdam Science Park 904 1098 XH Amsterdam Netherlands k.i.e.olsson@uva.nl; b Advanced Research Center for Nanolithography Science Park 106 1098 XG Amsterdam Netherlands

## Abstract

Defect engineering offers an important route to property tuning in hard coatings for advanced applications. Transition metal nitrides, such as CrN, are widely used for their mechanical resilience, but their nitrogen-rich analogue CrN_2_ remains poorly understood, especially at the atomic scale. This study employs density functional theory to investigate the energetics and how intrinsic defects (vacancies, interstitials, and anti-sites) and extrinsic impurities (hydrogen and oxygen) influence the structural, electronic, magnetic, and mechanical response of CrN_2_, in comparison to the more commonly studied CrN. With directional N–N bonding and semiconducting character, CrN_2_ shows high sensitivity to defect incorporation, including local spin polarisation, gap states, and mechanical softening. In contrast, CrN's metallic character enables effective screening of similar defects, preserving its structural, magnetic, electronic, and mechanical integrity. Hydrogen induces anisotropic distortions and mechanical degradation in CrN, while oxygen enhances hardness. These findings reveal how defect chemistry and bonding anisotropy govern mechanical performance, with implications for the design and optimisation of chromium nitride-based coatings.

## Abstract 2

DFT reveals that CrN_2_ is highly defect sensitive, with vacancies and impurities causing changes in electronic structure and mechanical softening, while CrN remains more tolerant. Insights guide defect-engineered hard coating design.

## Graphical abstract

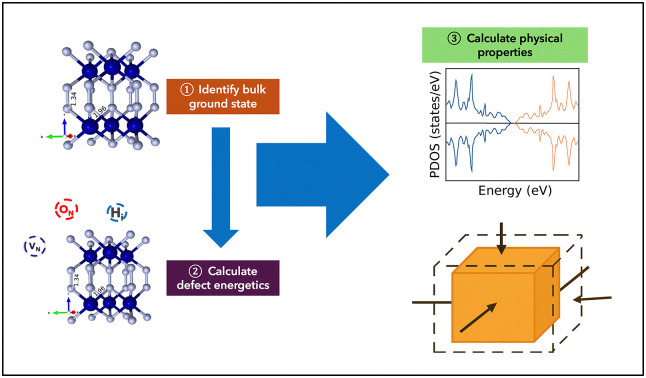

## 1 Introduction

Chromium nitrides (Cr_*x*_N_*y*_) are a class of binary transition-metal nitrides (TMNs) well-known for hardness,^1–3^ resistance to wear and corrosion,^4–8^ high melting point,^9^ thermal stability,^10^ and electrical stability,^11^ with superior surface oxidation properties as compared to other TMNs.^12,13^ These properties make them suitable as hard protective coatings,^14–16^ biomedical implants,^17^ cutting tools,^18^ in steel alloys,^19^ and engine components.^20^ Despite their intrinsic material advantages, the overall performance of many TMNs is influenced by the presence and nature of atomic-scale defects. Synthesis of stable sub-stoichiometric TM mononitrides has been reported for TiN,^21–24^ VN,^25,26^ and CrN.^27–30^ The recent discovery of nitrogen rich transition metal pernitrides (TMN_2_), which exhibit exceptional mechanical properties such as ultra-incompressibility and high hardness, has expanded the class of materials considered for use in industrial hard coatings.^31–37^ Whilst research has focused on these materials' stability and mechanical properties, understanding of the presence and effects of defects within TM pernitrides remains unexplored. Moreover, the performance of these materials as protective coatings is further influenced by the material's interaction with impurities like hydrogen and oxygen that can be present during their growth and become trapped at intrinsic defects sites, or absorbed from the atmosphere.^38,39^ These atomic impurities are particularly critical because they can lead to degradation mechanisms such as embrittlement,^40^ compromising the material's performance and longevity. Therefore, it is crucial to understand how intrinsic and extrinsic point defects (impurities) influence the physical properties of CrN-based materials.

Several experimental and theoretical studies have reported changes in structural, electronic and mechanical properties in the presence of defects in TMNs. Experimental studies supported by density functional theory (DFT) calculations showed structural changes in MoN^41^ where the lattice parameter was reduced with a decrease in cation to anion ratio, and in HfN^42^ where the lattice distorted from cubic to rhombohedral. Similarly, changes in electronic properties such as the introduction of new states around the Fermi level was observed in ScN through experimental^43^ and theoretical^44–46^ works, in TiN experimentally^47^ and theoretically^22,38,48,49^ and in VN experimentally.^26,50^ In terms of mechanical properties, an increase in the concentration of nitrogen vacancies was experimentally observed to reduce the elastic moduli of group 4 TMNs such as TiN_*x*_ and ZrN_*x*_,^22,51–53^ while in HfN_*x*_, the moduli first increase for 0.8 ≤ *x* ≤ 1.0, and then decrease for 1.0 ≤ *x* ≤ 1.2.^42^ The opposite trend was observed for group 5 TMNs, where both VN_*x*_ and NbN_*x*_ have shown an increase in the elastic moduli.^53,54^ Of the group 6 TMNs, studies show that vacancies stabilize the mechanically unstable MoN_*x*_^55,56^ and WN_*x*_.^57^ Yet, similar investigations of defect-property relationship into chromium-based nitrides remain limited, making the role of stoichiometry in determining their physical properties an open question.

Due to its structural stability and magnetic ordering, the chromium mononitride phase (CrN) has been investigated widely as a key candidate for both fundamental studies and practical applications among Cr_*x*_N_*y*_. CrN undergoes a magneto-structural transition, adopting a paramagnetic cubic (*Fm*3̄*m*) structure at room temperature and transitioning to an antiferromagnetic orthorhombic (*Pnma*) phase near the Néel temperature (273–283 K).^27,58–61^ The experimentally measured mechanical hardness of CrN varies significantly (≈11–26 GPa) depending on several factors including method of synthesis, microstructure, and residual stress.^1,7,62,63^ Similarly, theoretical predictions reproduce this spread in hardness, largely influenced by the choice of computational method and hardness model.^64,65^ In the presence of intrinsic defects, a combined experimental and theoretical study revealed that increasing nitrogen vacancies reduces the lattice constants, shifts the bonding character, and enhances metallic behaviour in cubic CrN.^30^ A theoretical study^66^ showed that nitrogen point defects, in the form of vacancies and interstitials, can close the non-zero band gap in semiconducting cubic CrN. This contrasts with experimental reports of pristine CrN exhibiting metallic behaviour across the transition and showing decreased resistivity below the Néel temperature.^58,67,68^ In the orthorhombic phase, intrinsic defects, including chromium and nitrogen vacancies, antisites, and interstitials, alter the electronic structure by introducing shallow states near the Fermi level, modifying d and p orbital distributions, inducing spin polarization, and disrupting antiferromagnetic order.^69^ Extrinsic defects such as oxygen incorporation increase CrN's hardness^70,71^ but the effect of hydrogen remains unexplored.

Whilst the mononitride phase has been extensively studied in the literature, the pernitride phase remains largely unexplored. The pernitride phase – CrN_2_ crystallizes in the *P*6_3_/*mmc* space group and features an anti-NiAs type structure. It consists of CrN_6_ triangular prisms stacked along the *c*-axis through rigid N–N dimers, which contribute to its axial incompressibility.^37^ Early calculations suggested a Vickers hardness of approximately 46 GPa, indicating potential superhard characteristics.^72^ However, subsequent analyses reported an ideal shear strength around 30 GPa,^73^ which is below the expected threshold for superhard materials. While Vickers hardness reflects resistance to plastic deformation from indentation, ideal shear strength provides a theoretical upper limit for shear deformation in defect-free crystals. The difference between the two highlights the role of atomic bonding and deformation mechanisms in governing hardness. These discrepancies underscore the need for comprehensive evaluations when assessing material hardness. Moreover, experimental approaches often face challenges in isolating and characterizing specific defects at the atomic level, making it difficult to establish a direct correlation between individual defects and their effect on material properties.

To further understand the role of intrinsic defects and atomic impurities in the mechanical robustness of chromium nitride-based coatings, we focus on the less-explored pernitride CrN_2_. CrN is included primarily as a well-studied reference to validate our approach. We also extend its study by examining the link between defects and mechanical properties, which has not been systematically addressed before. Both phases are previously identified as mechanically hard, with theoretically predicted Vicker's hardness values in the range typical for protective applications (below 40 GPa).^64,74^ The present work is designed as a dilute-limit baseline, where isolated point defects are examined to establish their energetics and associated changes in electronic and mechanical properties. Most previous theoretical studies of CrN have focused on the energetics of intrinsic defects, especially nitrogen vacancies, since these are central to stoichiometry and stability in thin films.^30,66^ Here, we extend beyond this scope by performing size-converged dilute-defect calculations and systematically linking defect energetics to electronic structure and, crucially, mechanical response. While CrN serves as a benchmark, no prior work has to the best of our knowledge addressed point defects in CrN_2_. Our work therefore provides the first systematic study of how its directional N–N bonding governs defect energetics, induces local spin polarisation, and drives mechanical softening. By analysing both intrinsic defects (vacancies, anti-sites, interstitials) and extrinsic impurities (hydrogen and oxygen), we identify how defects modify elastic moduli and hardness, thereby influencing the durability of mononitride and pernitride phases. These insights highlight CrN_2_ as a defect-sensitive, but potentially tunable nitride, and provide guidance for optimising chromium nitride-based coatings.

## 2 Methodology

To identify chromium nitride phases relevant for hard-coating applications, we investigated different compositions (CrN_2_, Cr_3_N_4_, CrN, Cr_3_N_2_, and Cr_2_N) and their polymorphs from the Materials Project^75^ selecting only structures within 0.5 eV per atom of the convex hull for further evaluation. The bulk formation energy, *E*^bulk^_f_,^76^ was calculated using:1
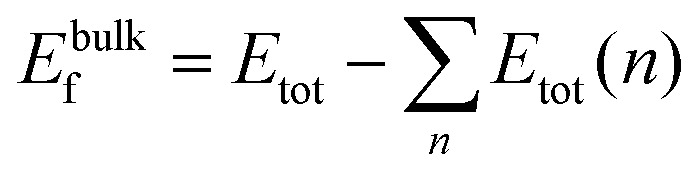
where *E*_tot_ is the total energy of the compound and *E*_tot_(*n*) represents the total energies of the elemental references, AFM bulk Cr and an isolated N_2_ molecule. All calculations were conducted within the DFT framework using the Vienna *ab initio* simulation package (VASP version 6.5.1).^77–79^ The Perdew–Burke–Ernzerhof (PBE) functional^80^ with the generalized gradient approximation (GGA)^81^ and the projector-augmented wave (PAW) method^82,83^ were used to accurately model electronic exchange–correlation interactions and electron–ion interactions in our DFT calculations. To describe the strong on-site Coulomb interactions due to Cr 3d electrons in CrN, we applied the Dudarev approach with a Hubbard *U* term, *U*_eff_,^84,85^ in alignment with previous literature.^86^ A *U* term was not applied to CrN_2_; benchmark calculations with *U* = 1 eV showed only minor shifts in the placement of defect induced states without altering their qualitative nature and has been included in the SI (Fig. S9). In the absence of experimental data to fit a reliable *U* parameter, applying such corrections would risk introducing arbitrary bias. Moreover, to remain consistent with previous theoretical studies of CrN_2_,^72,73^ we have employed the GGA functional for this material. After convergence tests on conventional unit cells (with convergence criteria of 1 meV per atom) the plane wave energy cutoff was set to 770 eV and a *Γ*-centered *k*-point mesh of 16 × 16 × 16 for CrN and 14 × 14 × 5 for CrN_2_, with Methfessel–Paxton smearing^87^ with a width of 0.1 eV. Electronic convergence was achieved with energy changes below 10^−6^ eV, and ionic relaxation continued until forces on atoms were below −0.01 eV Å^−1^. Different magnetic configurations, including antiferromagnetic (AFM), ferromagnetic (FM), and a paramagnetic-like ordering within the framework of SQS-DLM, were tested for cubic CrN. FM was chosen for further analysis as an approximation to the room-temperature paramagnetic state. A test on a nitrogen vacancy shows that while absolute formation energies vary modestly (≤0.3 eV) between FM, AFM and PM-like descriptions, the relative defect stability is unaffected (see SI, Section S1).

To assess the mechanical properties, elastic constants were calculated using the energy-strain method as implemented in VASPKIT.^88^ Mechanical stability of these structures was assessed according to the Born–Huang stability criteria.^89,90^ Elastic properties were derived using the Voigt approximation, from which bulk modulus (*K*_V_), Young's modulus (*E*_V_), and shear modulus (*G*_V_) were obtained. Polycrystalline averages were then used to evaluate bulk-relevant mechanical metrics, including Poisson's ratio (*ν*), Pugh's ratio (*k*), the universal elastic anisotropy index (*A*^U^), and Vickers hardness (*H*_V_) estimated using Tian's empirical model.^64^

Defects were introduced into relaxed, pristine structures and subsequently geometry optimised. For defect formation energy convergence, supercell sizes from 2 × 2 × 2 (48 atoms) to 4 × 4 × 2 (192 atoms) were tested for CrN_2_ and 2 × 2 × 2 (64 atoms) to 4 × 4 × 4 (512 atoms) for CrN. Convergence was defined by a change in defect formation energy of less than 0.02 eV between successive supercells (for the full range see Table S3 in the SI). To minimize size effects while maintaining computational efficiency, a 3 × 3 × 2 supercell (108 atoms) for CrN_2_, and a 3 × 3 × 3 supercell (216 atoms) was selected for CrN. The corresponding *k*-point meshes were 2 × 2 × 2 for CrN and 5 × 5 × 3 for CrN_2_. All defect calculations were performed in the dilute-limit, *i.e.* one defect per size-converged supercell (216 atoms for CrN, 108 atoms for CrN_2_). This corresponds to effective concentrations of ≈0.5–0.9 at% of the sublattice, sufficient to capture isolated-defect behaviour while minimizing defect–defect interactions. Inequivalent vacancy and antisite defect sites were identified using the site-occupation disorder (SOD) code,^91^ while interstitials were located *via* Atomsk^92^ or identified by symmetry-based crystallographic sites (tetrahedral, octahedral) from the Bilbao Crystallographic Server.^93^ The defect formation energy, *E*^def^_f_(*q*), is defined as:^94^2
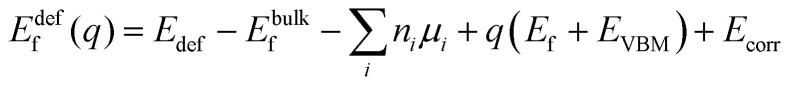
where *E*_def_ is the total energy of the defective cell, *E*_bulk_ is the total energy of the pristine cell, and *μ*_*i*_ is the chemical potential of species *i* added or removed. *E*_f_ represents the Fermi energy relative to the valence band maximum, *E*_VBM_. All defect energetics reported herein follow Kröger–Vink notation.^95^ The stability of point defects was evaluated under both Cr-rich and N-rich conditions.^76^ These conditions were defined through chemical potentials of Cr (*μ*_Cr_) and N (*μ*_N_), which vary depending on the growth environment. Under Cr-rich conditions *μ*_Cr_ is set equal to the chemical potential of bulk Cr, while *μ*_N_ is derived from the equilibrium condition *μ*_Cr_ + *μ*_N_ = *μ*_CrN_, where *μ*_CrN_ is the chemical potential of bulk CrN. Conversely, under N-rich conditions, *μ*_N_ is set equal to half the chemical potential of molecular nitrogen gas 
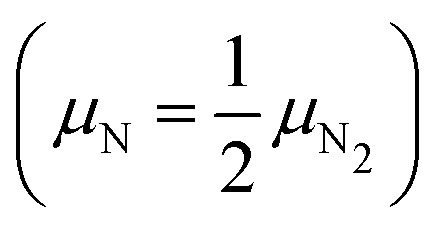
, and *μ*_Cr_ is again determined by equilibrium with bulk material. For materials with a band gap, a correction term *E*_corr_ was applied to address electrostatic interactions between charged defects and their periodic images. The Kumagai–Oba scheme,^96^ as implemented in Spinney^97^ was employed, as it refines the Freysoldt, Neugebauer, and Van de Walle (FNV) approach^98–100^ by using anisotropic point-charge energy and atomic-site potentials for alignment. This is particularly important for systems like CrN_2_, which exhibit anisotropic dielectric tensors due to their non-cubic symmetry. For CrN_2_, which has a calculated band gap of 0.73 eV, the dielectric constant was calculated *via* density functional perturbation theory (DFPT) as *ε*_*xx*_ = 26.085 and *ε*_*zz*_ = 22.378, allowing for defect-related changes in physical properties to be assessed for the most stable configurations. For metallic CrN, only neutral defects were considered, since efficient electronic screening renders charged states physically irrelevant within the defect formalism.^100^ Accordingly, all analysis in the main text is restricted to neutral defects, providing a consistent, Fermi-level-independent basis for comparing local bonding and electronic structure across systems.

## 3 Results

**Fig. 1:**
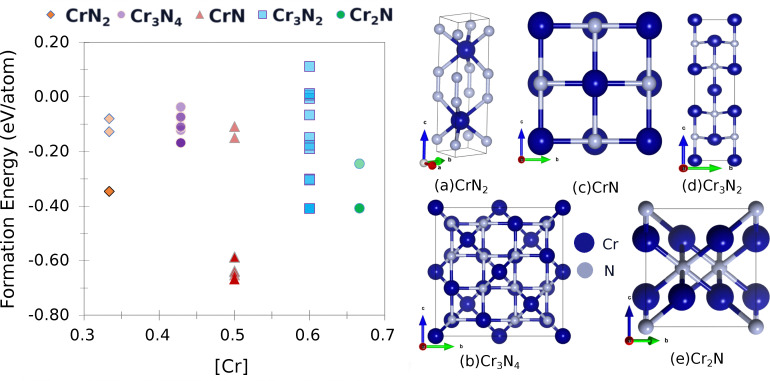
(Left) Formation energy for varying chromium concentration in Cr_*x*_N_*y*_ and (right) structural representations of (a) CrN_2_ (b) Cr_3_N_2_ (c) CrN (d) Cr_3_N_2_ (e) Cr_2_N.

The structural and physical properties of Cr_*x*_N_*y*_ were initially examined across five stoichiometric compositions to assess trends in stability and defect behaviour (Fig. 1). Among these, CrN (*x* = 1, *y* = 1) is known to be the most thermodynamically stable phase, consistent with previous reports,^65,101^ and it thus serves as a reference point in this study. CrN_2_, by contrast, lies higher in formation energy (Δ*E* = 0.241 eV) but is the only composition exhibiting superior mechanical properties as compared to CrN. These two phases were therefore selected for detailed defect analysis.

Defects lead to lattice distortions, changes in the electronic landscape, and mechanical properties. In this work, we focus on the pernitride CrN_2_, examining the effects of dilute-limit intrinsic defects and extrinsic impurities (oxygen and hydrogen) on its structural, electronic, and mechanical response. For comparison, the corresponding behaviour in CrN is also evaluated, providing a well-established reference. The defects considered are grouped as follows: (i) chromium defects: vacancy, antisite, and interstitial, (ii) nitrogen defects: vacancy, antisite, and interstitial, (iii) hydrogen defects: substitution and interstitial, and (iv) oxygen defects: substitution and interstitial. The most probable defects are then identified from their formation energies, and their impact on the physical properties is compared to the pristine phases.

### 3.1 Defect energetics, structure, and magnetic response

The lowest defect formation energies (*E*^def^_f_(*q*)) for various defects, calculated using eqn (2), are presented in Table 1. These correspond to the most stable configurations, identified by sampling multiple atomic sites and charge states across the Fermi level. The results are shown for both N-rich (Cr-poor) and N-poor (Cr-rich) chemical potential limits, with the Fermi level fixed at the valence band maximum (VBM). Results of alternative configurations are provided in Table S4 in the SI.

**Table 1:** *E* ^def^_f_ values of the most stable defects in Cr_*x*_N_*y*_. All other investigated defect positions are included in the Table S4 of the SI

Composition	Defect	N-poor (eV)	N-rich (eV)
CrN_2_	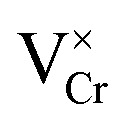	6.10	5.06
	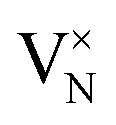	1.82	2.34
	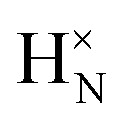	0.30	1.50
	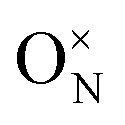	−0.46	0.06

CrN	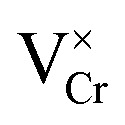	3.30	2.13
	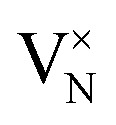	0.54	1.71
	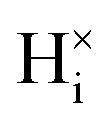	1.17	1.17
	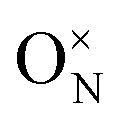	−3.39	−2.81

In CrN_2_, 
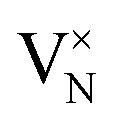
 is the most favourable intrinsic defect, with a neutral formation energy of 2.34 eV, as compared to 1.71 eV in CrN. The higher value in the pernitride reflects the disruptive effect of removing an N atom from the dimerised bonding network, which is absent in CrN. Nitrogen anti-sites and N interstitials in CrN_2_ are highly unfavourable (*E*^def^_f_(*q*) > 5 eV), consistent with the tight bonding environment in the dimerized structure. CrN, in contrast, stabilizes a split N interstitial at 3.41 eV, which, in a previous work is calculated to be 3.77 eV using different methodologies.^66^ Most Cr-related defects in both systems exhibit high formation energies (>5 eV), with the exception of Cr vacancy which in CrN_2_ is unfavoured as compared to in CrN where V_Cr_ = 2.13 eV suggests moderate feasibility.

Substitutional extrinsic defects show stronger chemical potential dependence. In both CrN_2_ and CrN, substitutions at the chromium site, 
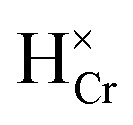
 and 
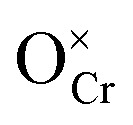
, are energetically unfavourable (*E*^def^_f_(*q*) > 5 eV), whereas N-site substitutions and some interstitials are stable. In CrN_2_, hydrogen substitution in a nitrogen site with 
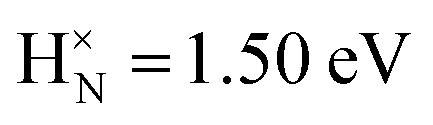
 is the lowest defect configuration. Two low-energy interstitial H configurations at *C*_3v_ symmetry yield values of 2.58 eV and 2.92 eV (figures included in SI, Fig. S4). In CrN, the most stable H defect is the interstitial site 
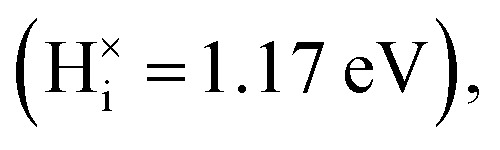
 followed by the bond-centred site (1.38 eV) and the tetrahedral site (2.32 eV). Hydrogen substitution at the N site 
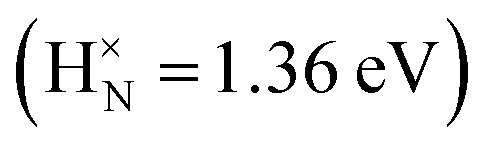
 is also thermodynamically accessible. Oxygen incorporation in CrN_2_, with 
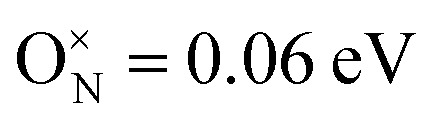
 is only marginally costly underscoring the susceptibility of the pernitride lattice to oxygen impurities. In CrN, the same substitution is strongly favourable with 
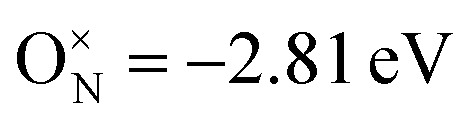
, indicating a strong thermodynamic driving force due to the greater stability of Cr–O bonds compared to Cr–N.^102^ Interstitial oxygen illustrates some disparity: while all configurations in CrN_2_ are unfavourable (*E*^def^_f_(*q*) > 5 eV), CrN can stabilize three interstitial geometries, including a split interstitial (*E*^def^_f_(*q*) = 1.35 eV, *C*_s_ symmetry), and two higher-energy sites (3.47 and 3.91 eV), illustrated in the SI (Fig. S4).

**Fig. 2:**
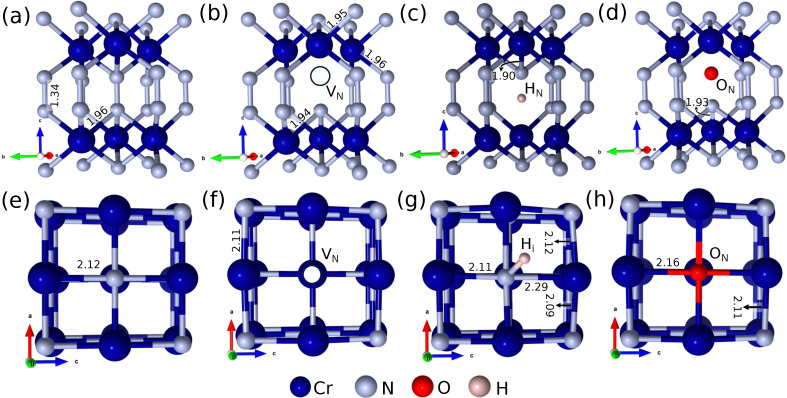
Overview of structural distortions in presence of defects, (a)–(d) show the pristine, 
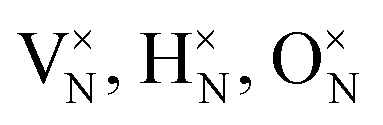
, respectively in CrN_2_ and (e)–(h) show the pristine, 
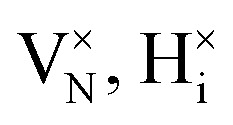
 and 
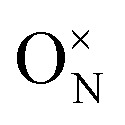
 in CrN. Bond lengths reported are in Å.

Point defects perturb the local bonding environment, introducing structural distortions that often couple with changes in charge and spin distribution. Fig. 2 presents relaxed structures of the lowest-energy neutral point defects, with bond length distributions provided in the SI (Fig. S5 and S6). In CrN_2_, a nitrogen vacancy 
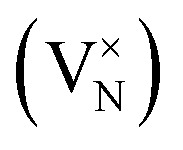
 leads to slight bond contraction (0.01–0.02 Å) in nearby Cr–N and N–N pairs, attributed to the removal of electronic repulsion from the missing N atom. In contrast, substitutional hydrogen 
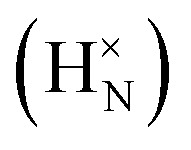
 and oxygen 
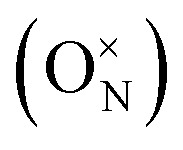
 defects cause surrounding Cr and N atoms to relax outward. In CrN, 
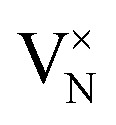
 pushes nearby Cr atoms outward and N atoms inward, consistent with metallic bonding and delocalized electrons. Oxygen substitution 
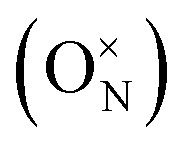
 causes asymmetric relaxation, with Cr moving away and N moving closer, while remaining aligned with the original N site. H_i_ occupies off-centre positions, displacing adjacent Cr and N atoms due to its small size and high mobility.

CrN_2_ exhibits a non-magnetic ground state in its pristine form. Upon introduction of a 
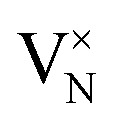
 defect, a total magnetic moment of 0.375*μ*_B_ emerges, predominantly on defect adjacent Cr atoms due to defect-induced electronic localisation. The origin and nature of these spin-polarised states are analysed in detail in the subsequent section. The 
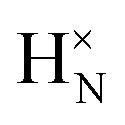
 defect does not alter the magnetic character, whereas 
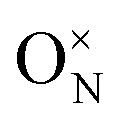
 introduces a total magnetic moment of −1.041*μ*_B_, again centred on nearby Cr atoms. This indicates a strong coupling between defect type, local structure, and spin polarization. In CrN, each Cr atom exhibits a magnetic moment of 2.97*μ*_B_, and this local moment persists across all defect configurations examined. Although no significant net magnetization change is observed, the spin density redistributes locally, indicating defect-induced polarization without quenching the overall magnetic ordering.

These results reveal that the energetics and structural signatures of point defects in CrN_2_ are highly sensitive to both defect chemistry and bonding environment, as a consequence of its directional N–N bonding. In contrast, CrN exhibits greater structural rigidity and tolerance to point defects.

### 3.2 Electronic properties

**Fig. 3:**
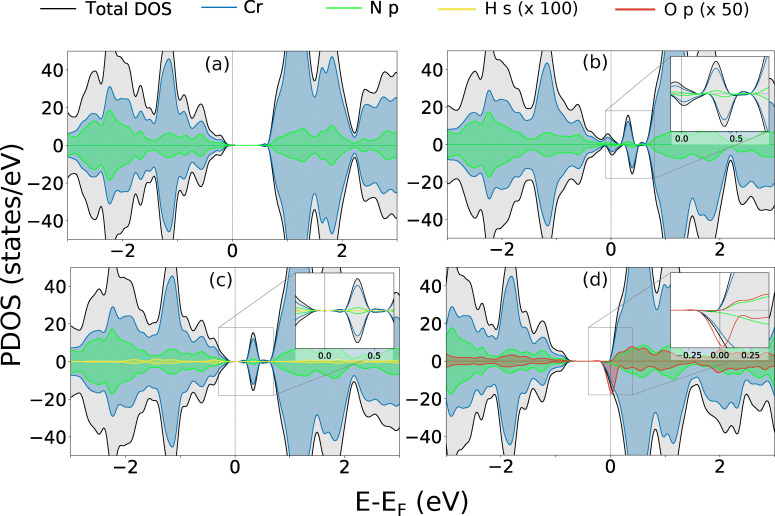
Projected density of states of (a) pristine (b) 
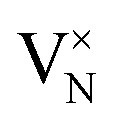
 (c) 
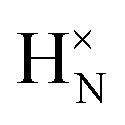
 (d) 
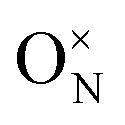
 in CrN_2_. Insets highlight the defect-induced in-gap states.

Having identified the most stable dilute-limit defects under equilibrium conditions, we now examine their influence on the electronic structure of CrN_2_ through projected density of states (PDOS) (Fig. 3). Additionally, Bader charge analysis (Table 2), is employed to quantify local redistribution of electron density around the defect sites and to investigate charge transfer and bonding effects. CrN_2_ in its pristine form (Fig. 3a) exhibits a spin-symmetric landscape with a calculated band gap of 0.73 eV which falls within the range of previously calculated theoretical works (between 0.5 and 1.14 eV).^72,73^ By contrast, the PDOS of CrN, shown in SI Fig. S3 reflects a ferromagnetic ordering, with a metallic spin-up channel and a partial gap in spin-down (half-metallicity). Bader charge analysis reveals a charge transfer of 1.39*e* from each Cr atom in CrN_2_, distributed across two neighbouring N atoms, indicating mixed ionic-covalent bonding. This is slightly lower than in CrN, where a charge transfer of 1.43*e* occurs to one N, reflecting more ionic character.

**Table 2:** Bader charges (*q*_i_, i = Cr, N, O, H) for the pristine and defective compositions of Cr_*x*_N_*y*_. *q*_i_ for the defective structures refers to the the nearest neighbours species around the defect site of the lowest energy neutral defects. The full range of Bader charges is provided in Table S5 in the SI

System	*q*_Cr_ (e)	*q*_N_ (e)	*q*_O,H_ (e)
CrN_2_			
Pristine	1.39	−0.63, −0.76	
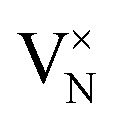	1.30	−1.04	
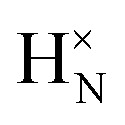	1.25	−1.09	0.24
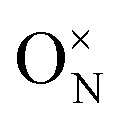	1.40	−0.53	−0.79
CrN			
Pristine	1.43	−1.43	
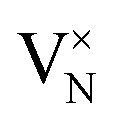	1.27	−1.46	
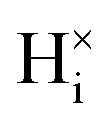	1.54	−1.50	0.27
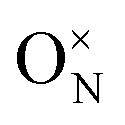	1.50	−1.45	−1.39

**Fig. 4:**
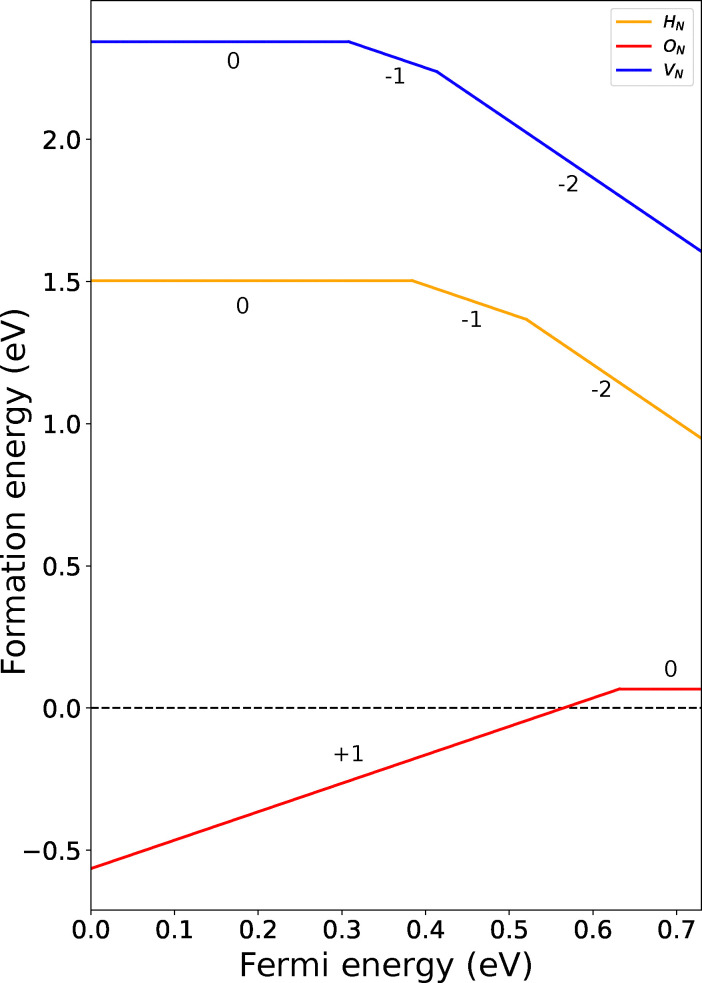
Charge transition level (CTL) diagram of the lowest energy defects under N-rich chemical potential in CrN_2_.

In CrN_2_, the introduction of a nitrogen vacancy 
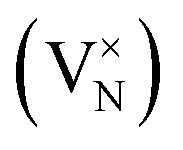
 leads to under-coordination of adjacent Cr atoms, resulting in localized states within the band gap consisting of Cr d-orbitals with minor N p-character (Fig. 3(b)). These states are strongly spin-polarized, reflecting broken spin symmetry due to electron localization. Bader charge analysis supports this, revealing local charge accumulation on the Cr atoms (*q*_Cr_ = 1.30*e*) surrounding the vacancy. The neighbouring N atom exhibits a more negative Bader charge (*q*_N_ = −1.04*e*), indicative of partial retention of charge from the disrupted N–N dimer. The unpaired electron localizes primarily on Cr sites, reinforcing the observed d-dominated in-gap states. 
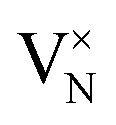
 exhibits two charge transitions at Fermi levels of 0.31 eV and 0.41 eV, corresponding to formation energies of 2.65 eV 
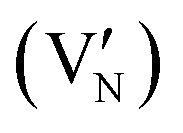
 and 3.06 eV 
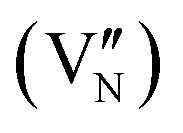
, respectively as seen in Fig. 4. In CrN, 
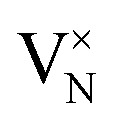
 (Fig. S7(b)) in CrN leads to a redistribution of electronic states around the Fermi level without opening a gap. A reduction in the Bader charges on nearby Cr atoms from 1.43*e* to 1.27*e* is seen while N atoms largely retain their original charge (1.43*e*), with slight increases to 1.46*e* observed on atoms further from the defect. Despite this local reorganization, the overall metallic character is preserved. This suggests that the defect is efficiently screened, highlighting CrN's more delocalized, metallic bonding framework and its relative electronic rigidity compared to CrN_2_.

The ability of hydrogen to alter the band gaps of semiconducting nitrides has been observed in several works,^103–105^ and the ease with which hydrogen can incorporate in a material makes it a common impurity. In CrN_2_, 
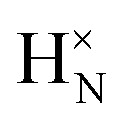
 introduces shallow occupied states near the conduction band edge (Fig. 3(c)), accompanied by a slight upward shift in the Fermi level. The Bader charge value on H is 0.24*e*, reflecting partial electron donation to nearby Cr atoms that accommodate the excess charge (*q*_Cr_ = 1.25*e*). No deep in-gap states are observed, indicating that 
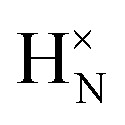
 behaves as a shallow donor in its neutral state. The minimal spectral weight of H s-orbitals suggests weak hybridization and reduced bonding involvement compared to nitrogen, implying that Cr–H interactions are more ionic in nature. This points to hydrogen substitution as a means of tuning carrier concentrations through Fermi level modulation in CrN_2_. 
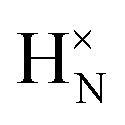
 also exhibits multiple charge states with transition levels at *E*_F_ = 0.38 and 0.52 eV with 
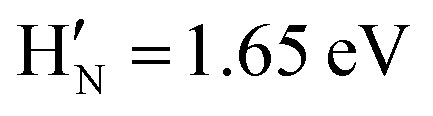
 and 
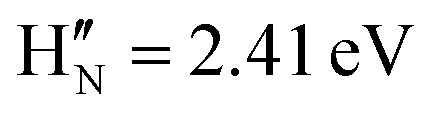
 (Fig. 4). In CrN, H_i_ is efficiently screened (Fig. S7(c)), with no discrete in-gap states and only a modest increase in Cr 3d weight below the Fermi level. This is further confirmed by partial donation of charges by H_i_ (+0.27*e*). Nearest neighbour Cr becomes more oxidised (+1.54*e*) and N becomes slightly more negative (−1.50*e*) indicating a local but delocalised perturbation.

The electronic structure of CrN_2_ in the presence of an oxygen atom substituted on a nitrogen site 
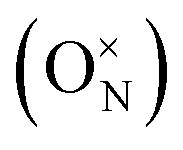
 is shown in Fig. 3(d). 
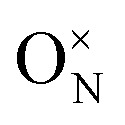
 in CrN_2_, leads to a small increase in electron accumulation at the oxygen site (−0.79*e*). This is accompanied by a redistribution of charge within the local environment where the nearest neighbouring Cr atom becomes slightly more oxidised (+0.10*e*) and the neighbouring N becomes less negative by a comparable amount (+0.10*e*), indicating a broader shift in electron density beyond the immediate substitution site. The PDOS reveals hybridisation of Cr d-states and O p-orbitals in the spin-down channel near the conduction band, alongside a clear shift of the Fermi level toward the conduction band edge. This defect stabilizes a +1 charge state with formation energy 
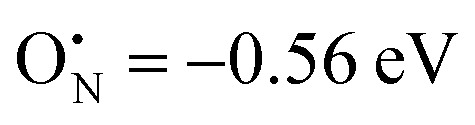
, as observed in Fig. 4. This transitions from the +1 state to the neutral state with 
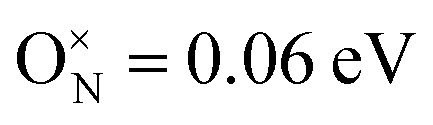
 at *E*_F_ = 0.63 eV. In CrN, 
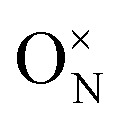
 withdraws local charge due to its higher electronegativity, carrying −1.39*e versus* −1.43*e* for N in the pristine lattice. This oxidises neighbouring Cr (up to +1.50*e vs.* +1.43*e*), while N atoms remain largely unchanged (−1.43*e* to −1.45*e*). Thus, 
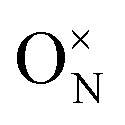
 perturbs the local electronic environment but does not disrupt the overall magnetic state, unlike in CrN_2_.

These results highlight the contrasting defect responses of CrN_2_ and CrN, closely tied to their defect energetics and magnetic response discussed in the previous section. In CrN_2_, defects create localized, spin-polarized states that modulate the electronic structure and magnetism, whereas in CrN they are more delocalized, and preserved metallicity. These differences reflect the underlying bonding environments and electronic rigidity of the two systems.

### 3.3 Mechanical properties

**Fig. 5:**
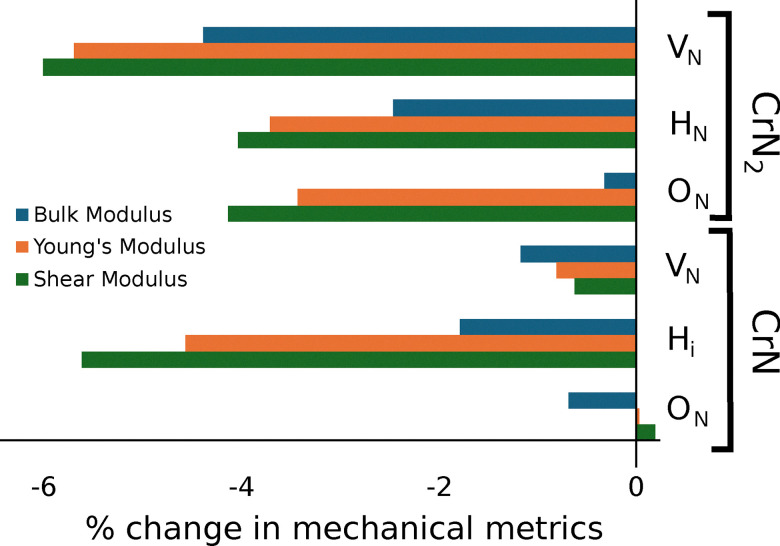
Percentage change in bulk modulus, Young's modulus, and shear modulus for the most stable point defects in CrN_2_ and CrN, relative to their pristine values.

With the energetic and electronic behaviour established, we assess the impact of the most stable defects on elasticity and hardness relative to bulk values (Table 3). To better visualize the trends, Fig. 5 summarizes the percentage changes in bulk (*K*_V_), Young's (*E*_V_), and shear moduli (*G*_V_) for the most stable defects in both CrN_2_ and CrN. Full second-order elastic tensor components (*C*_*ij*_) for both pristine and defected structures are provided in SI (Subsections 1.1 and 2.1). The Born–Huang criteria confirm that all of the defective structures discussed are mechanically stable.

**Table 3:** Calculated bulk modulus (*K*_V_), Young's modulus (*E*_V_), Shear modulus (*G*_V_), Vicker's hardness (*H*_v_), Pugh's ratio (*k*), Poisson's ratio (*ν*) and universal elastic anisotropy (*A*^U^) of the most stable defects in CrN_2_ and CrN

Crystal structure	*K*_V_ (GPa)	*E*_V_ (GPa)	*G*_V_ (GPa)	H_v_ (GPa)	*k*	*ν*	*A* ^U^
CrN_2_							
Pristine	337.45	593.79	246.04	31.67	1.37	0.21	0.64
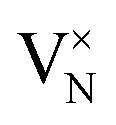	322.70	560.03	231.28	29.72	1.39	0.21	0.55
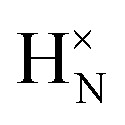	329.17	571.75	236.16	30.20	1.39	0.21	0.59
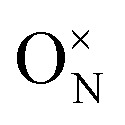	336.36	573.61	235.90	29.41	1.43	0.22	0.63
CrN							
Pristine	237.76	412.87	170.53	23.98	1.40	0.21	0.05
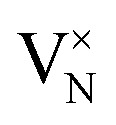	233.97	409.55	169.48	24.14	1.39	0.21	0.05
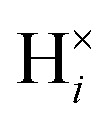	235.82	394.09	161.32	21.84	1.46	0.22	0.02
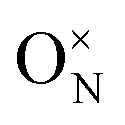	236.15	413.07	170.90	24.26	1.38	0.21	0.05

Pristine CrN_2_ exhibits higher elastic moduli than CrN, with average *K*_V_ = 337.45 GPa, *E*_V_ = 593.79 GPa, and *G*_V_ = 246.04, compared to 237.76 GPa, 412.87 GPa and 170.53 GPa for CrN, respectively. These values are consistent with previous studies, where the bulk modulus of CrN_2_ has been reported to be around 325 GPa^73^ theoretically and 286 GPa^37^ experimentally,^73^ while CrN values are 241.33 GPa^86^ theoretically and 260 GPa^106^ experimentally. This difference reflects the directional bonding in hexagonal CrN_2_, particularly the presence of N–N dimers, which provide additional rigidity. In contrast, the cubic symmetry of CrN gives rise to a more isotropic elastic response, consistent with its delocalised, metallic bonding environment. This distinction underpins the differing sensitivities of each material to local perturbations.

The introduction of a single 
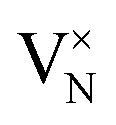
 leads to changes in the mechanical stiffness of CrN_2_ and CrN, with a stronger reduction in the former. In CrN_2_, *K*_V_ decreases from 337.45 GPa to 322.70 GPa (−4.37%), with corresponding decreases in both *E*_V_ from 593.79 GPa to 560.03 GPa (−5.68%) and *G*_V_ from 246.04 GPa to 231.28 GPa (−5.99%). This can be traced back to the disruption of its directional N–N bonding network, previously shown to be sensitive to local charge and spin rearrangements in previous sections. These reductions are accompanied by changes in directional stiffness but no symmetry breaking evident from Fig. S10 and S11 in the SI. In comparison, CrN shows less pronounced reductions with *K*_V_ decreasing from 237.76 GPa to 233.97 GPa (−1.6%). A similar result was reported in a previous work on MoN_*x*_, where the reduction was calculated to be 2%.^53^ This trend remains consistent in the case of *E*_V_ and *G*_V_, where both moduli show a decrease from the pristine value of 412.87 GPa to 409.55 GPa (−0.8%) and 170.53 GPa to 169.48 GPa (−0.62%), respectively. Tian's hardness (*H*_V_) follows a similar pattern: CrN_2_ softens (−6.2%), while CrN shows a slight increase (+0.7%), aligning more closely with experimental values.^107^ CrN thus remains largely isotropic in mechanical response. This indicates the intrinsic presence of such defects in CrN, consistent with its low defect formation energy (Table 1). These results show that CrN_2_, with its more anisotropic bonding structure, is more susceptible to defect-induced mechanical degradation than CrN.

The mechanical response of CrN_2_ and CrN to hydrogen-related point defects reveals distinct trends in both isotropic and directional hardness. In CrN_2_, 
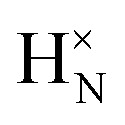
 leads to moderate reductions in *K*_V_ from 337.45 to 329.17 GPa (−2.5%), *E*_V_ from 593.79 to 571.75 GPa (−3.7%), and *G*_V_ from 246.04 to 236.16 GPa (−4.0%). *H*_V_ decreases by 4.7% (from 31.67 to 30.20 GPa), while Pugh's ratio slightly increases (from 1.37 to 1.39), and *A*^U^ drops from 0.64 to 0.59. This is linked to local lattice expansion and weakened Cr–H bonding, as suggested by the structural relaxation and partial charge transfer in Sections 3.1 and 3.2. Directional *E*_V_, shown in Fig. S10 in the SI, indicate uniform but mild softening across crystallographic planes, without significant symmetry disruption. In CrN, 
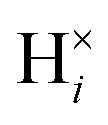
 reduces *K*_V_ from 237.76 to 235.82 (−0.82%), *E*_V_ from 412.87 to 394.09 GPa (−4.55%), *G*_V_ from 170.53 to 161.32 GPa (−5.4%), and *H*_V_ from 23.98 to 21.84 GPa (−8.9%). While *A*^U^ drops from 0.05 to 0.02, indicating an overall isotropic trend, directional moduli (Fig. S11) reveal symmetry changes of [001] in (001), deviations along^101^ in (011), and selective softening along [11̄0] in (111). Although the equilibrium solubility of hydrogen in these nitrides is very low (10^−7^−10^−9^ per site at 600–800 K and 1 bar H_2_), trapping at vacancies, dislocations, or interfaces can raise effective concentrations to levels comparable to our supercells.^108–110^ Experimental studies similarly show that hydrogen ingress degrades mechanical performance, with reduced hardness and adhesion loss in CrN coatings.^111^ This aligns with our prediction that an H-impurity causes softening. The softening we predict should thus be interpreted as the local elastic response near trapped H, providing a mechanistic link to hydrogen-induced embrittlement in both CrN_2_ and CrN.

The presence of a single 
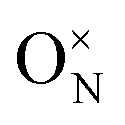
 in CrN_2_ reduces *K*_V_ from 337.45 to 336.36 GPa (−0.36%), *E*_V_ from 593.79 to 573.61 GPa (−3.42%) and *G*_V_ from 246.04 to 235.90 GPa (−4.15%). A more pronounced drop is observed in *H*_V_, which falls by over 7% from 31.67 to 29.41 GPa, accompanied by a slight decrease in *A*^U^ from 0.64 to 0.63. These changes arise from charge withdrawal and hybridisation of O-p and Cr-d states near the conduction edge (Section 3.2) which breaks the local N–N dimer bonding, reducing the different mechanical metrics. Despite these macroscopic effects, directional *E*_V_ plots remain nearly unchanged across most planes. A subtle asymmetry appears in the (101) plane, where the^102^ and [11̄01] directions no longer exhibit identical stiffness profiles, suggesting that oxygen induces weak but localised anisotropic distortions. In CrN, the mechanical response of 
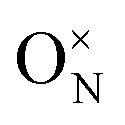
 is more subdued. *K*_V_ decreases from 237.76 to 236.15 GPa (−0.68%,) while both *E*_V_ and *G*_V_ increase by 0.04% and 0.2% respectively. *H*_V_ increases by 1.17%, implying enhanced resistance to plastic deformation. This behaviour reflects the formation of locally stronger, more ionic Cr–O bonds within CrN's metallic framework, which contributes to mild solid-solution strengthening. It further aligns with experimental observations of improved hardness of chromium oxynitride as compared to CrN.^71,112^ Directional *E*_V_ plots show no deviation from the pristine structure across all crystallographic planes, indicating that oxygen substitution induces minimal elastic perturbation in CrN's cubic lattice.

CrN_2_, though intrinsically harder than CrN, is more susceptible to impurity-induced softening particularly due to the disruption of directional N–N bonding. In contrast, 
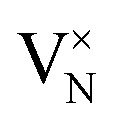
 and 
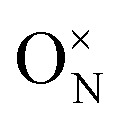
 cause minor changes in CrN's hardness with the latter slightly improving it. Hydrogen degrades both materials and introduces directional distortions *via* local bonding rearrangements. These findings suggest that while CrN_2_ may show superior initial mechanical metrics, CrN is likely to be more robust under non-ideal processing conditions or extended operational use where point defects and impurities are unavoidable.

## 4 Conclusions

Using density functional theory simulations, we investigated how intrinsic and extrinsic point defects influence the structural, electronic, and mechanical behaviour of two hard Cr–N phases: hexagonal CrN_2_ and cubic CrN. 
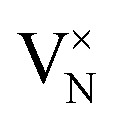
 is the most favourable intrinsic defect in both phases with diverging impact: in CrN_2_ they localise charge, introduce in-gap states, and cause pronounced elastic softening, whereas in CrN they are efficiently screened, leading to minimal changes. Hydrogen as 
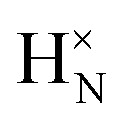
 in CrN_2_ also introduces in-gap states modestly and reduces stiffness, while in CrN, as 
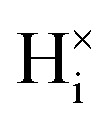
 breaks symmetry and drives anisotropic softening linked to embrittlement. 
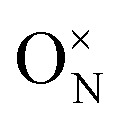
 in CrN_2_ induces spin polarisation and reduces stiffness, while in CrN it is well accommodated and even slightly enhances hardness. These results reveal that CrN_2_, despite its higher directional intrinsic hardness, is more defect sensitive due to its directional N–N bonding, whereas CrN is generally more tolerant but susceptible to hydrogen. This work provides the first systematic picture of defect-property relationships within the dilute-limit in CrN_2_ and demonstrates how defect control can be used to better understand Cr–N coatings for reactive and hydrogen-rich environments. Future work will extend this framework to explore multi-defect complexes and higher concentrations.

## Conflicts of interest

The authors declare no conflicts of interest.

## Supplementary Material

CP-027-D5CP02904J-s001

CP-027-D5CP02904J-s002

CP-027-D5CP02904J-s003

CP-027-D5CP02904J-s004

CP-027-D5CP02904J-s005

CP-027-D5CP02904J-s006

CP-027-D5CP02904J-s007

CP-027-D5CP02904J-s008

CP-027-D5CP02904J-s009

CP-027-D5CP02904J-s010

CP-027-D5CP02904J-s011

CP-027-D5CP02904J-s012

## Data Availability

The data supporting this article have been included as part of the supplementary information (SI). Supplementary information is available. See DOI: https://doi.org/10.1039/d5cp02904j. Additional computational data, including input parameters, processed structures, defect calculations, and relevant input/output files, are openly available on Zenodo (https://doi.org/10.5281/zenodo.16307517).
