# Supplementary figures and images for: Elucidating the impact of point defects on the structural, electronic, and mechanical behaviour of chromium nitride
Source: Phys Chem Chem Phys. 2025 Oct 7;27(42):22610–20. doi: 10.1039/d5cp02904j (PMC12517257; doi:10.1039/d5cp02904j)

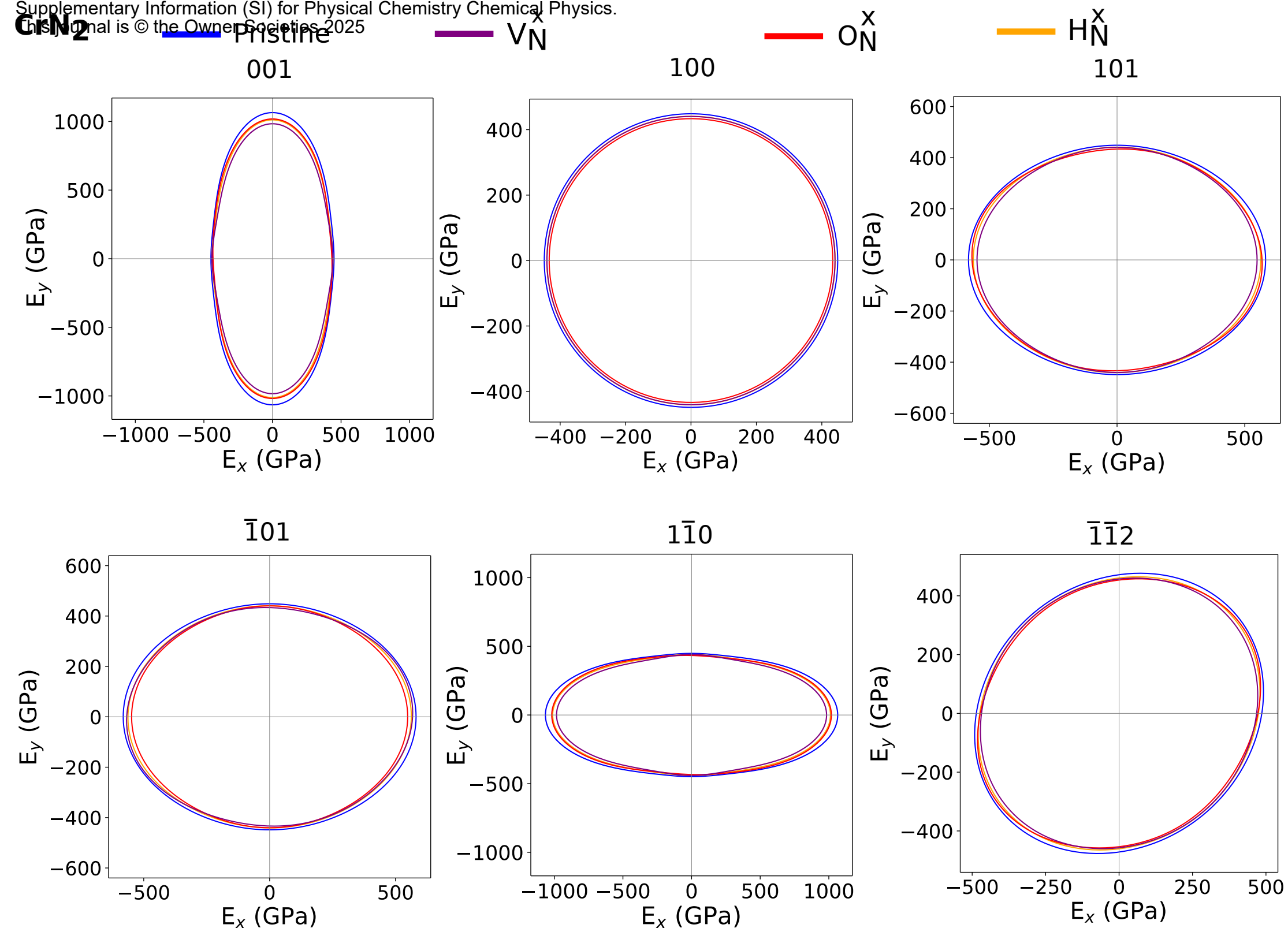

Supplement: CP-027-D5CP02904J-s001 [file CP-027-D5CP02904J-s001.pdf]

CrN<sub>2</sub>

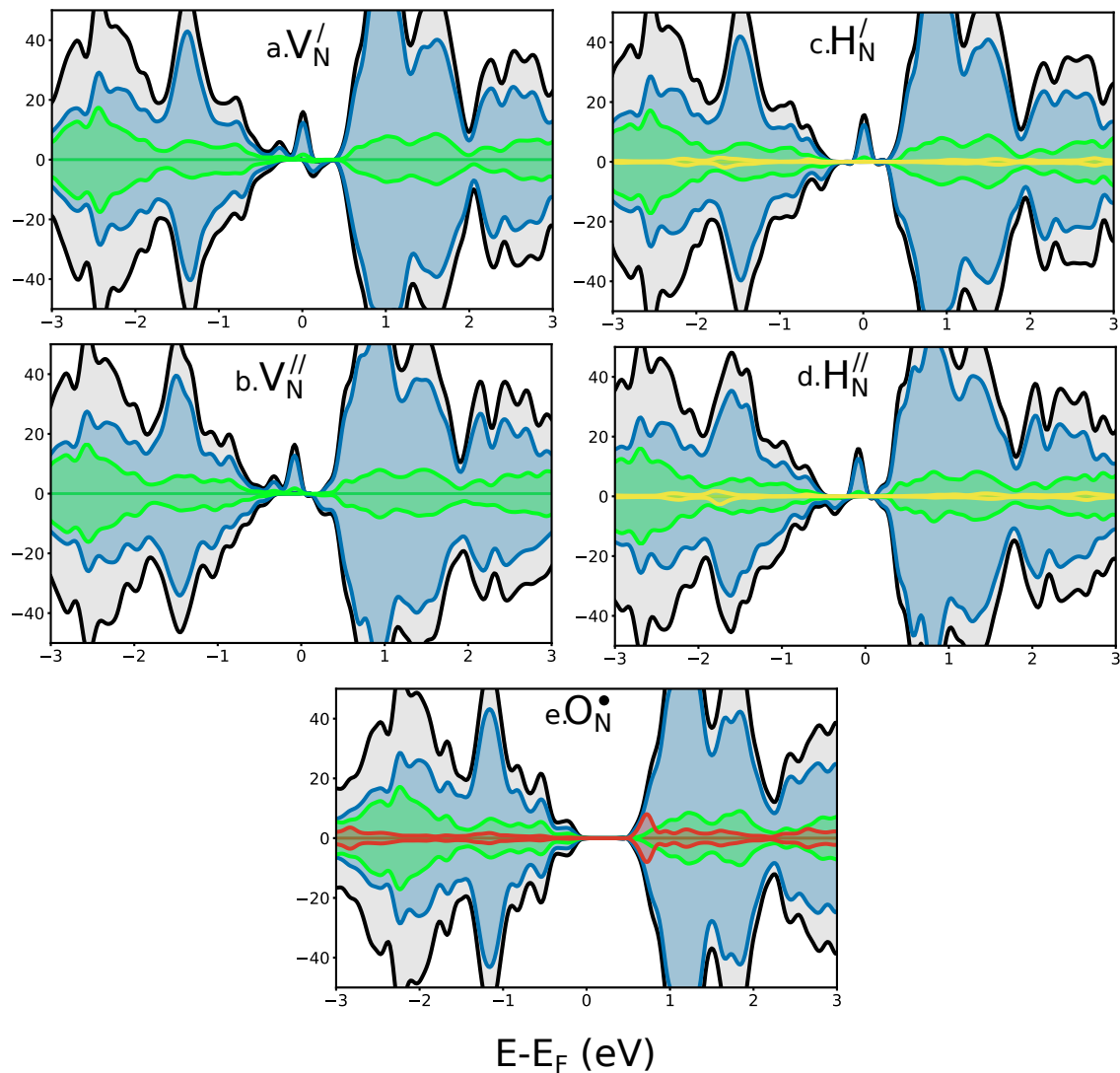

— Total DOS    — Cr d    — N p    — H s    — O p

Supplement: CP-027-D5CP02904J-s002 [file CP-027-D5CP02904J-s002.pdf]

PDOS (states/eV)

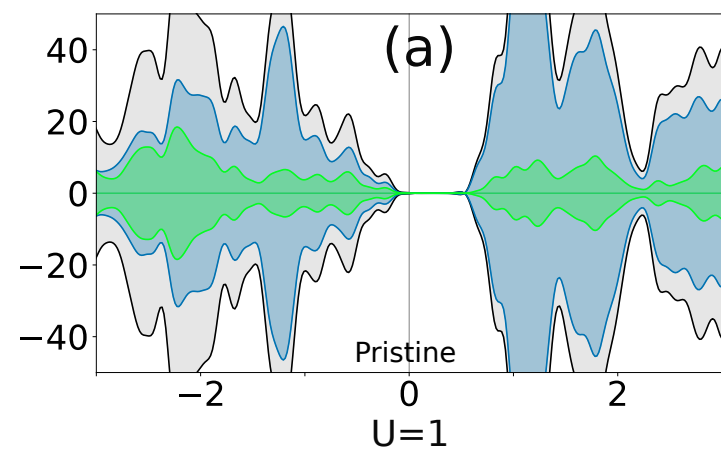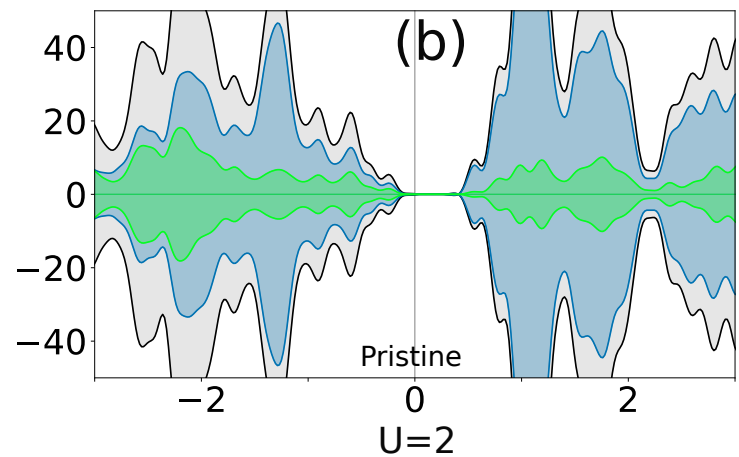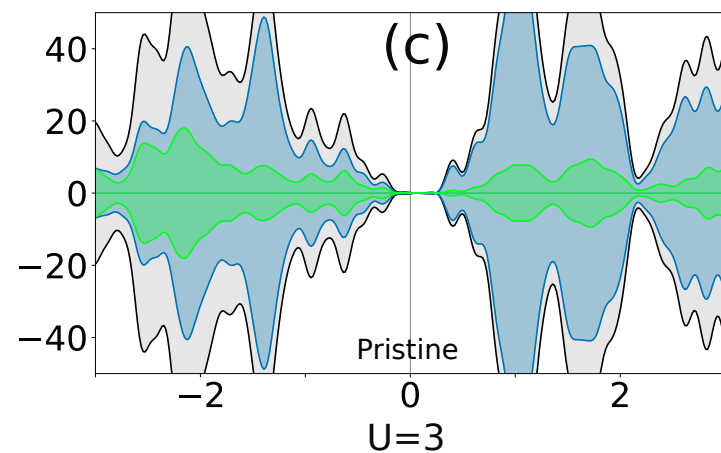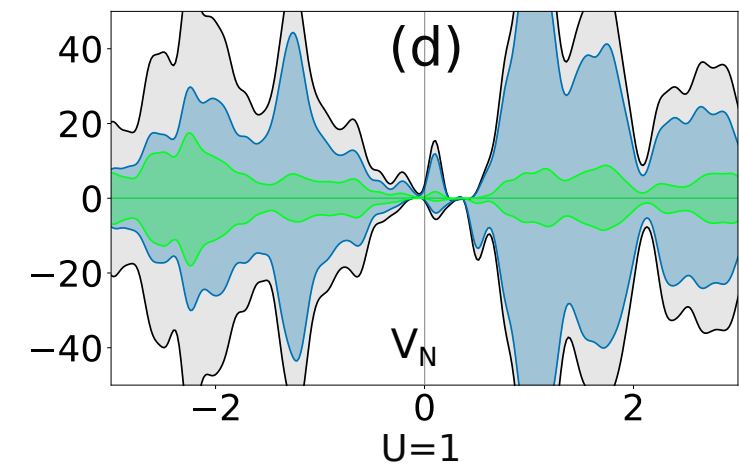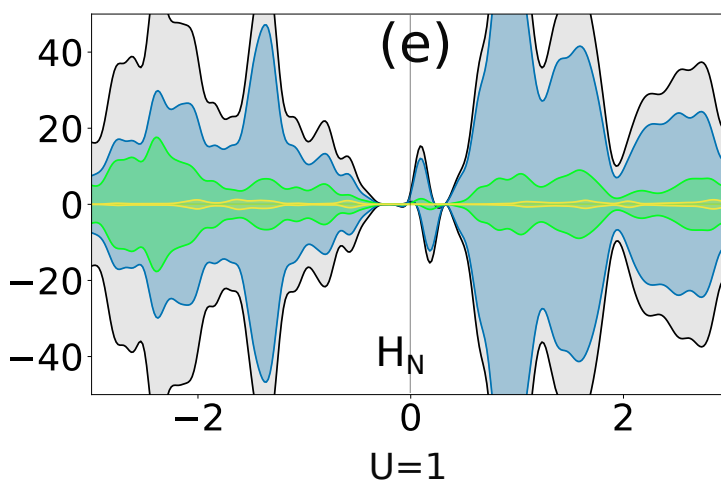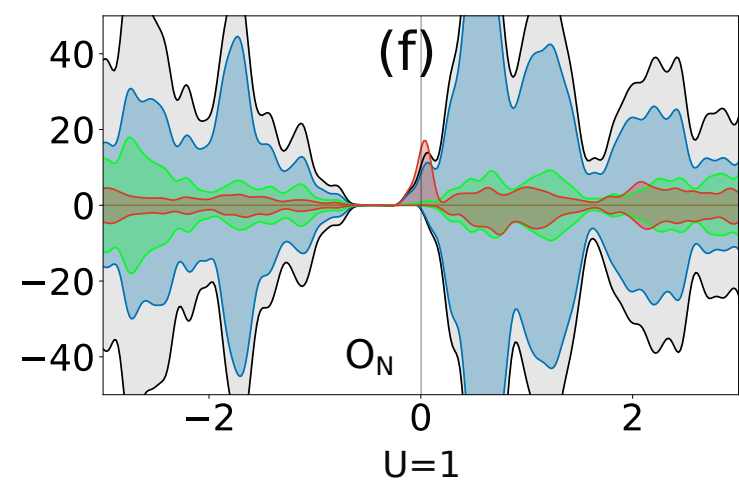

$E-E_F$  (eV)

Supplement: CP-027-D5CP02904J-s003 [file CP-027-D5CP02904J-s003.pdf]

—  $\text{Pristine}$ 
—  $\text{VN}^{\text{X}}$ 
—  $\text{ON}^{\text{X}}$ 
—  $\text{HN}^{\text{X}}$

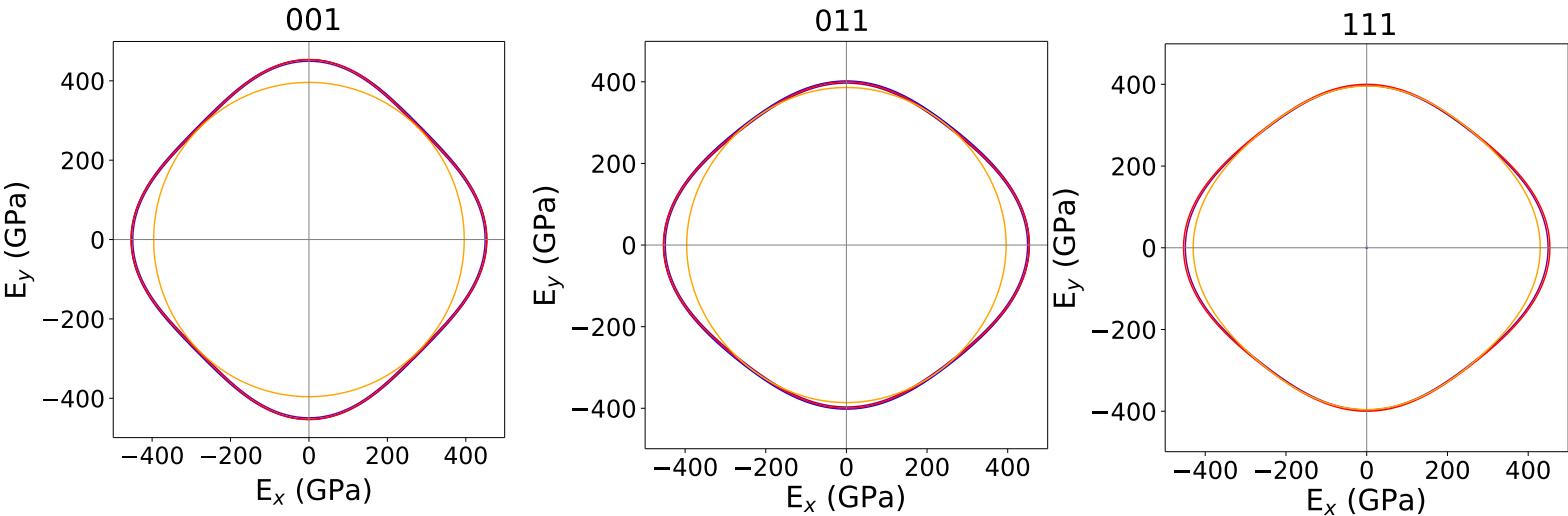

Supplement: CP-027-D5CP02904J-s005 [file CP-027-D5CP02904J-s005.pdf]

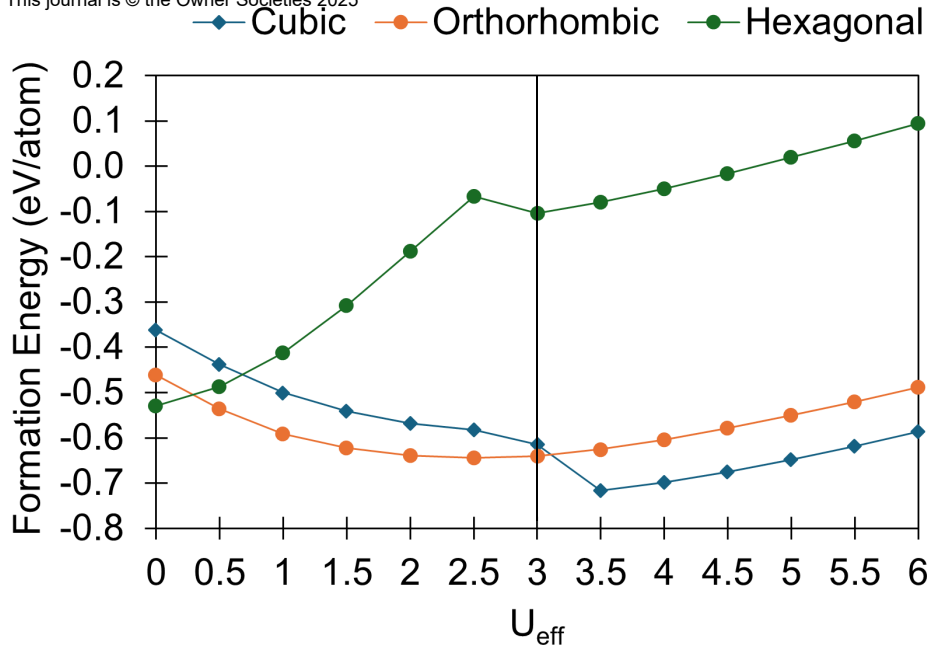

Supplement: CP-027-D5CP02904J-s006 [file CP-027-D5CP02904J-s006.pdf]

a.

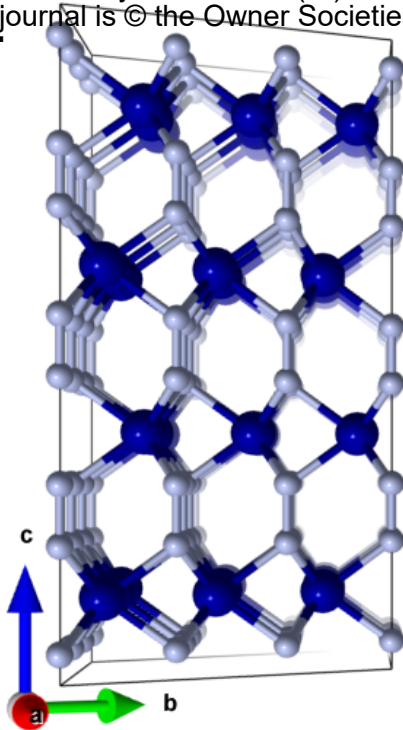

b.

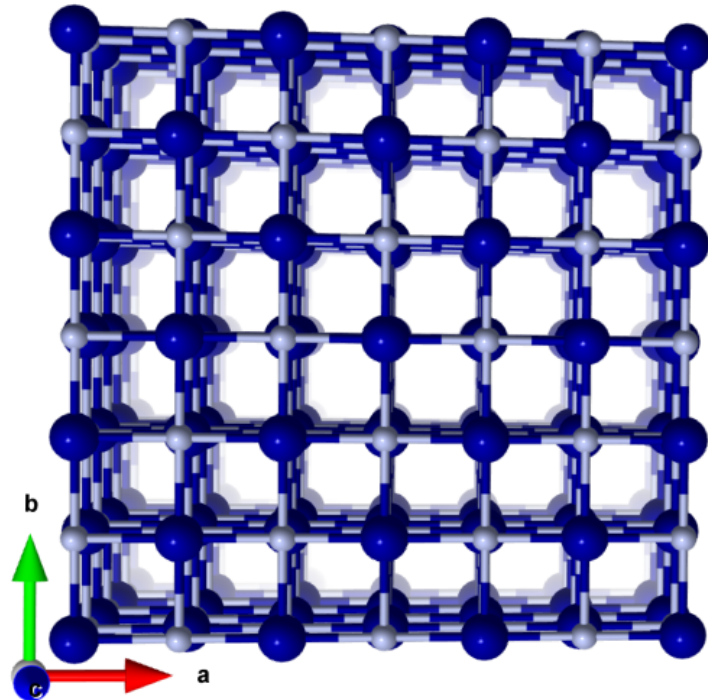

Supplement: CP-027-D5CP02904J-s007 [file CP-027-D5CP02904J-s007.pdf]

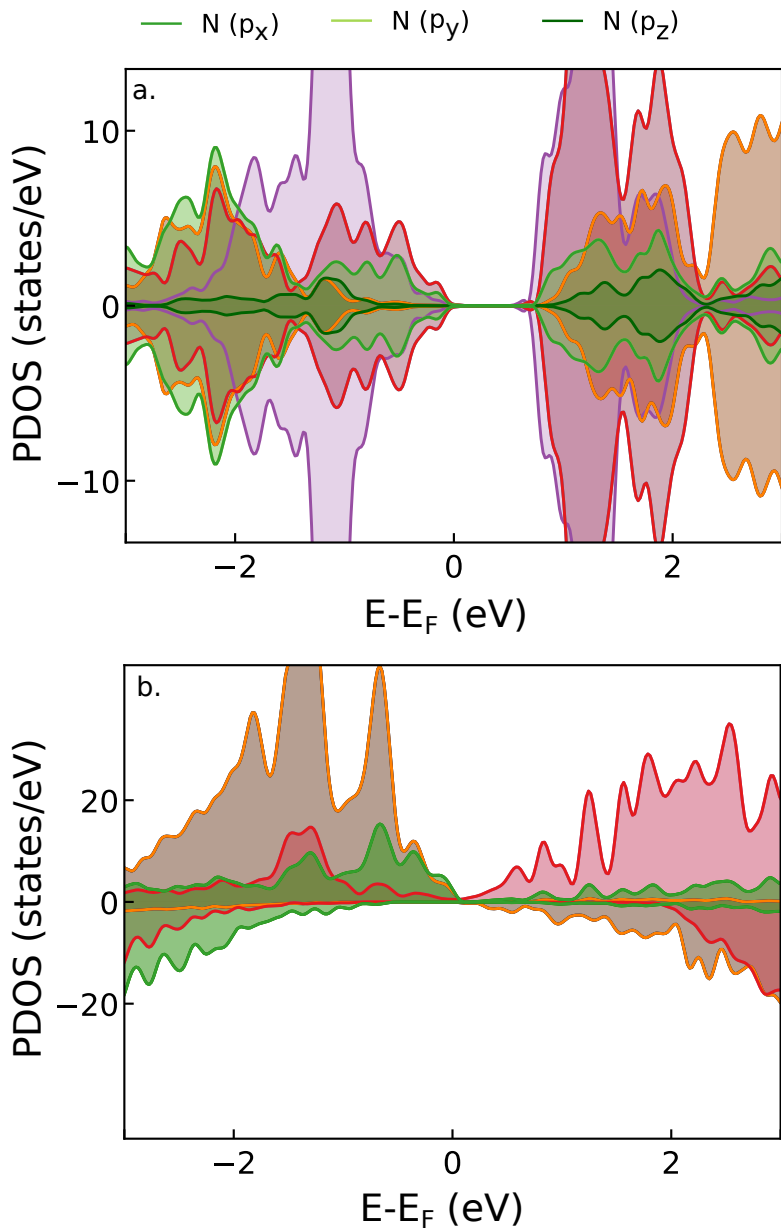

Supplement: CP-027-D5CP02904J-s008 [file CP-027-D5CP02904J-s008.pdf]

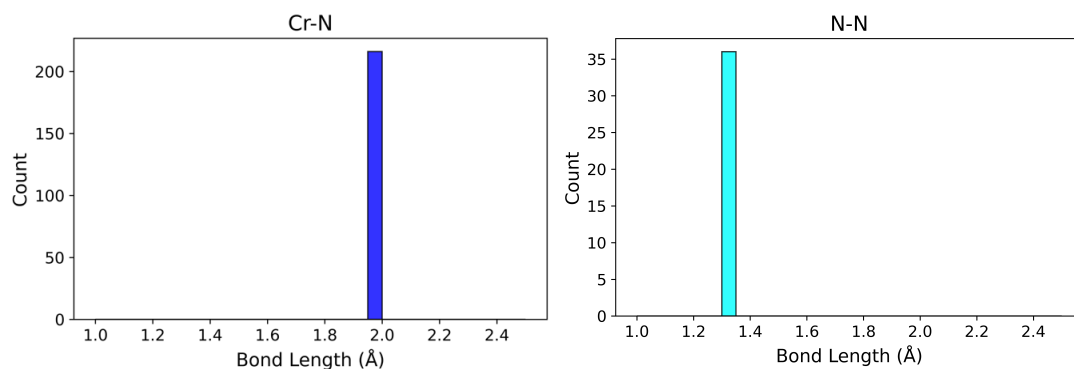

b.  $V_N^x$

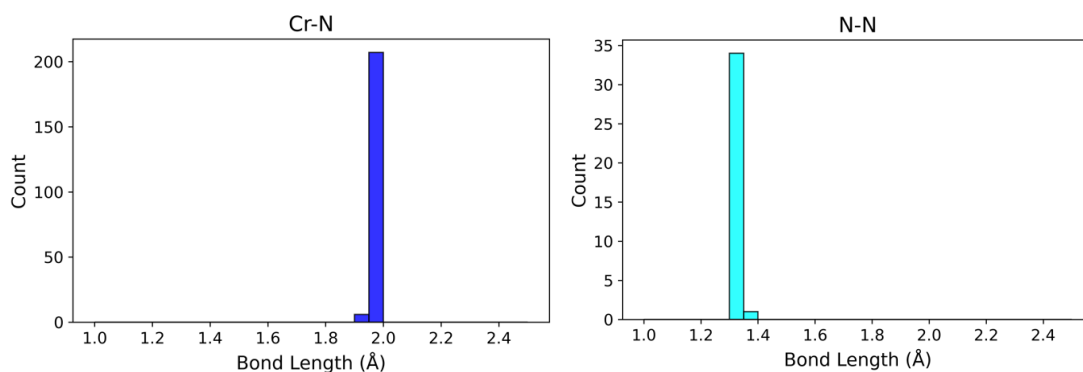

c.  $H_N^x$

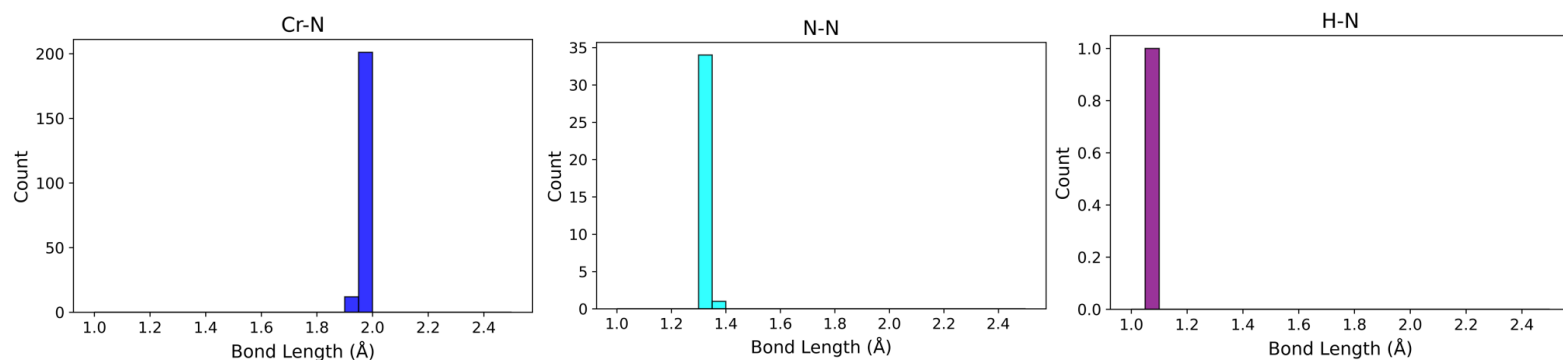

d.  $O_N^x$

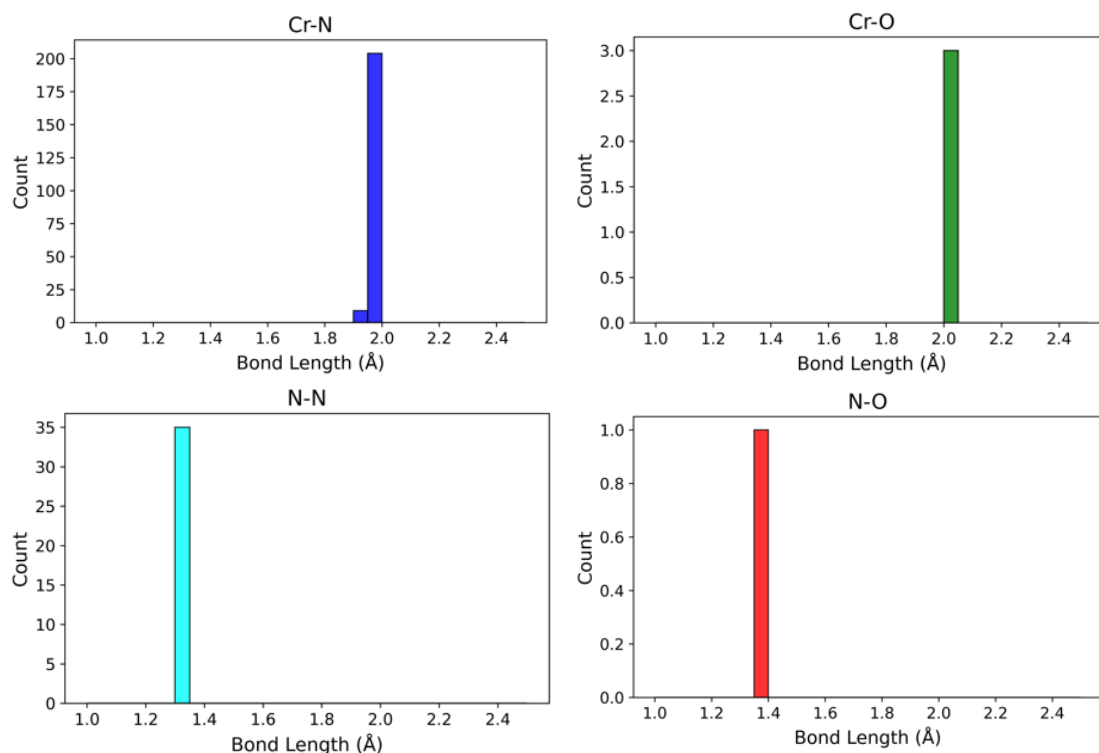

Supplement: CP-027-D5CP02904J-s010 [file CP-027-D5CP02904J-s010.pdf]

a. Pristine

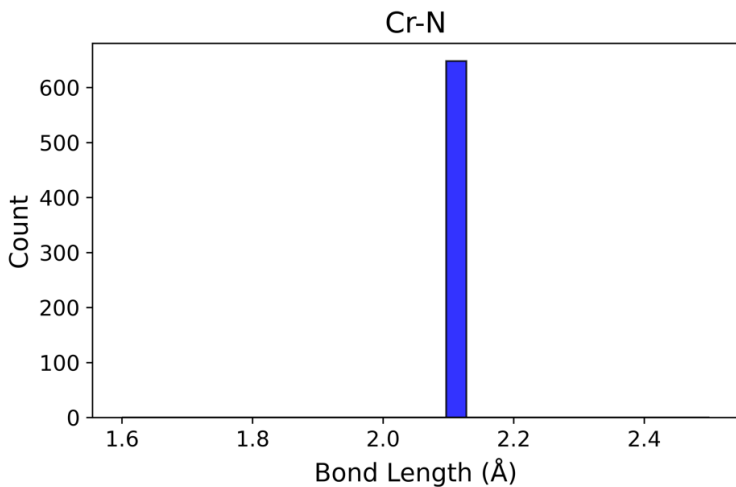

b.  $V_N^x$

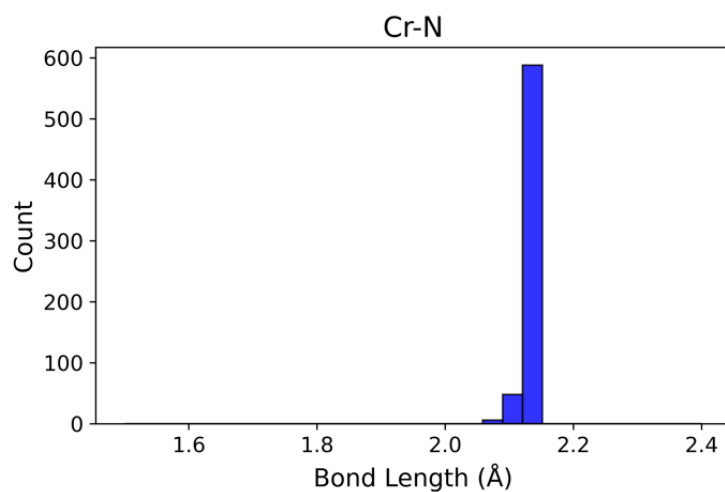

c.  $H_N^x$

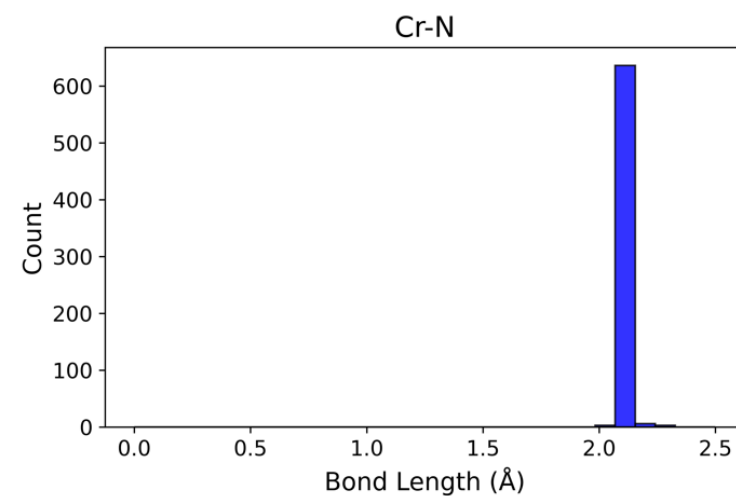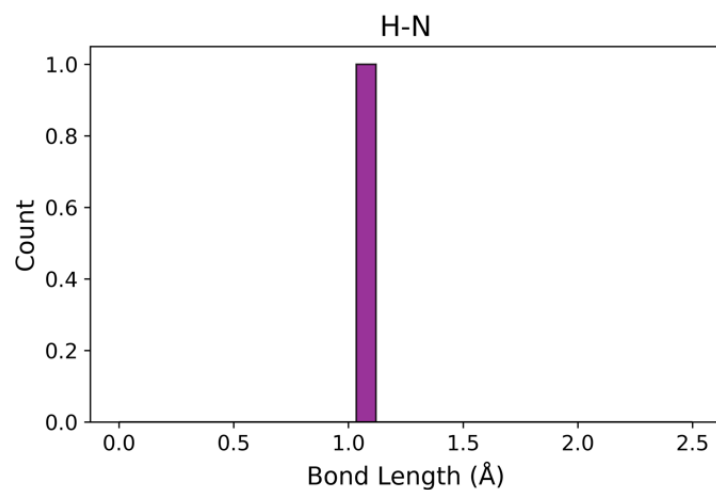

d.  $O_N^x$

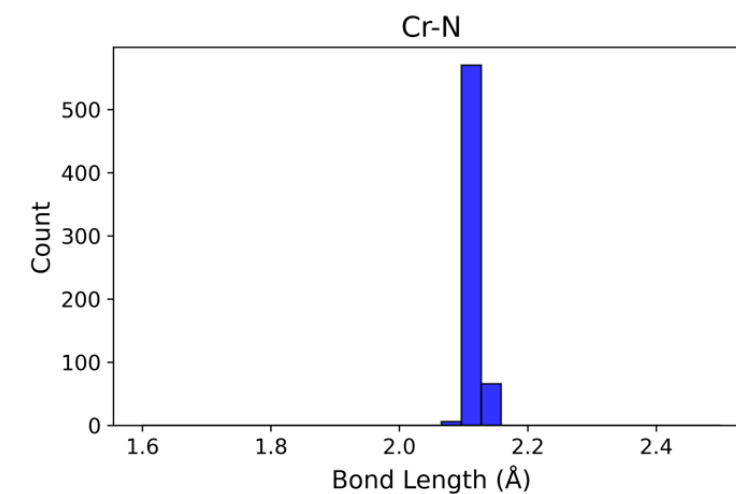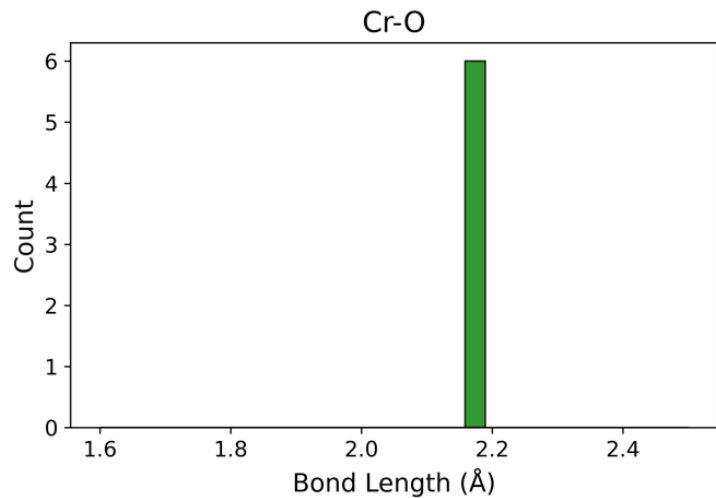

Supplement: CP-027-D5CP02904J-s011 [file CP-027-D5CP02904J-s011.pdf]

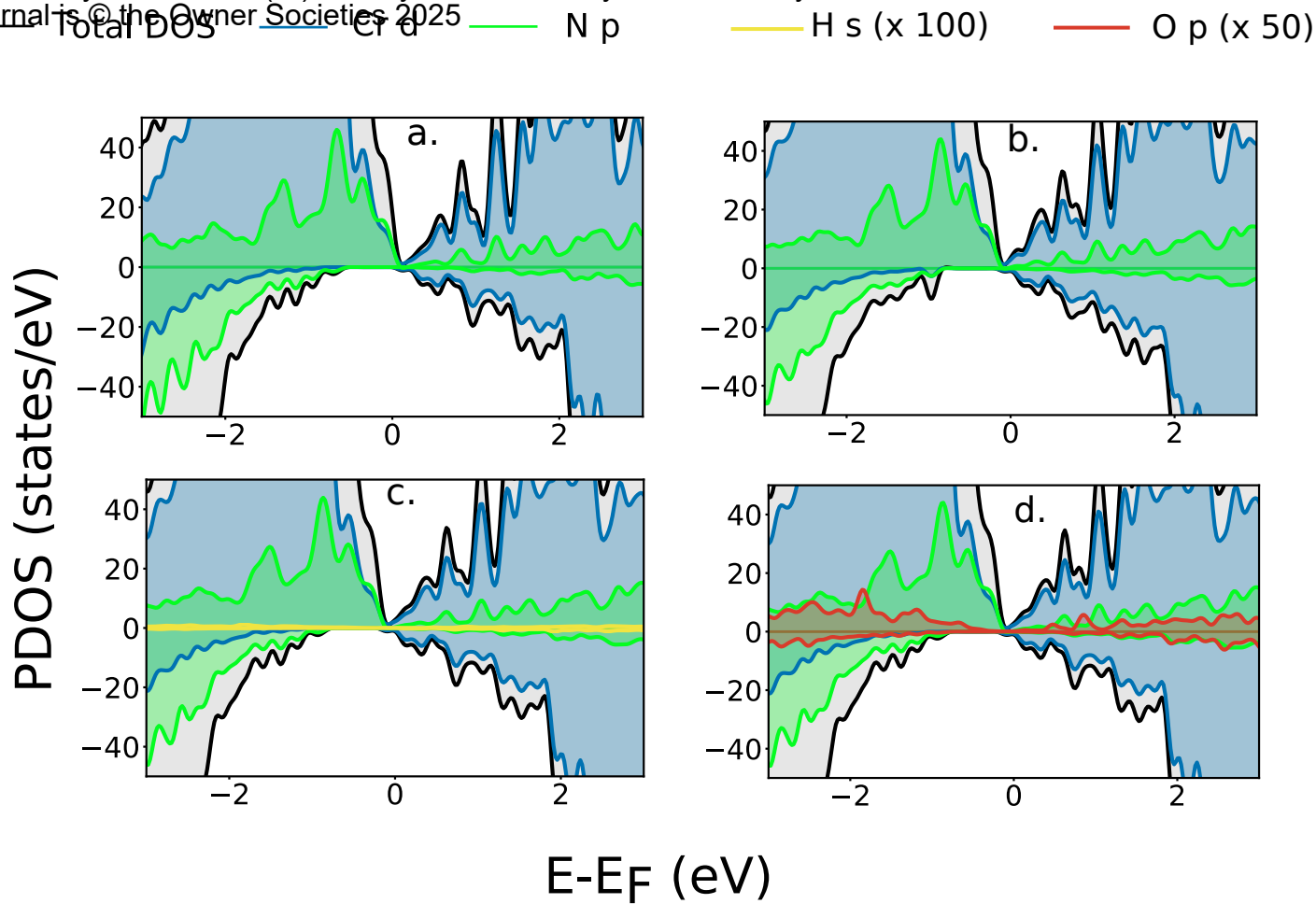

Supplement: CP-027-D5CP02904J-s012 [file CP-027-D5CP02904J-s012.pdf]
