# Supplementary material for: Elucidating the impact of point defects on the structural, electronic, and mechanical behaviour of chromium nitride
Source: Phys Chem Chem Phys. 2025 Oct 7;27(42):22610–20. doi: 10.1039/d5cp02904j (PMC12517257; doi:10.1039/d5cp02904j)
Supplement: CP-027-D5CP02904J-s004 [file CP-027-D5CP02904J-s004.pdf]

Supplementary Information (SI) for Physical Chemistry Chemical Physics.
This journal is © the Owner Societies 2025

Supplementary Information for "Elucidating the impact of point defects on the structural, electronic, and mechanical behaviour of chromium nitride"

Barsha Bhattacharjee [a,b], Emilia Olsson [a,b]
[a] *Institute of Theoretical Physics, University of Amsterdam, Science Park 904, 1098 XH Amsterdam, Netherlands.*
[b] *Advanced Research Center for Nanolithography, Science Park 106, 1098 XG Amsterdam, Netherlands.*

1 Bulk Characterisation

We conducted a systematic investigation into the effect of the Hubbard U parameter on the formation energy and physical properties of Cr_xN_y compositions, including CrN_2, Cr_3N_4, CrN, Cr_3N_2, and Cr_2N. Each polymorph was examined with multiple magnetic orderings-antiferromagnetic (AFM), ferromagnetic (FM), and spin-polarized non-magnetic (NM), to determine the ground state configuration.

In the mononitride CrN, formation energies and structural parameters were found to be sensitive to the value of U_{eff}. For $U_{\text{eff}} \geq 0.5\,\text{eV}$, the orthorhombic AFM ground state becomes energetically favorable, consistent with experimental and theoretical reports [1,2]. Lower U values tend to stabilize a hexagonal phase. The computed α angles agree well with experimental values: 88.23° [1] and 88.40° [3] correspond to $U_{\text{eff}} = 2$ and $2.5\,\text{eV}$, respectively, while $U_{\text{eff}} = 3\,\text{eV}$ yields $\alpha = 88.56°$. As the phase transition temperature is relatively insensitive to U [2], we adopt $U_{\text{eff}} = 3\,\text{eV}$ for consistency across all pristine and defective compositions.

While AFM orthorhombic CrN was consistently found to be the most stable configuration after structural relaxation, this study focuses on compositions relevant to hard coating applications, where the room-temperature cubic polymorph is of greater practical interest. The orthorhombic phase exists only below the Néel temperature and can be suppressed by synthesis techniques [4–6].

Within the cubic polymorph, we compared five magnetic configurations, namely FM and four AFM variants: AAFM, CAFM, GAFM, and AFM_{110} (Corliss-type). The AFM_{110} configuration exhibited the lowest formation energy (−0.66 eV/atom), followed by other AFM arrangements and FM (−0.58 eV/atom), with an energy difference of approximately 80 meV/atom. Despite the small energetic penalty, we selected the FM cubic CrN as our reference model because (i) it approximates the paramagnetic state relevant at room temperature [2], (ii) FM ordering describes cubic CrN structurally in agreement with experiments [7] whereas non-cubic lattice distortions are present in the AFM case (iii) mechanical properties calculated for both FM and AFM states show some differences (see Subsection 1.1); however, due to the significantly higher computational cost of AFM calculations, the FM configuration was adopted for all defect analyses.

Lattice distortions were observed in AFM configurations. AFM_{110} caused a distortion

in the γ angle, ranging from 90.1699° at $U - J = 0\,\text{eV}$ to 90.0328° at $U - J = 6\,\text{eV}$, consistent with magnetostructural coupling[8]. This distortion reflects a structural bridge between the cubic and orthorhombic phases via the α and γ angles. Elastic constants and other mechanical properties showed negligible variation across magnetic orderings.

Table S1: Magnetic moments (μB), lattice parameters (Å), and E_{form}(eV/atom) of Cr_xN_y as calculated with both GGA (no U) and GGA+U, U = 3 eV.

Composition	Method	Cr-N Bond Length (Å)	Cr Magnetic Moment (μB)	N Magnetic Moment (μB)	Lattice Parameter(Å)	E_{form} (eV/atom)
CrN_2	GGA	1.96	0	0	$a = 2.733$ $c = 7.382$	-0.36
CrN_2	Expt.[9]	2.00	-	-	$a = 2.747$ $c = 7.370$	-
CrN (FM)	GGA+U	2.12	2.97	-	$a = 4.25$	-0.59
CrN (AFM_{110})	GGA+U	2.11	±2.87		$a = 4.22$	-0.66
CrN	Expt.	2.07[10]	2.36[1]	-	$a = 4.15$[7]	-
Cr_2N	GGA	1.94	0	0	$a = 4.777$ $c = 4.400$	-0.407
Cr_2N	Expt.[11,12]	1.94[13]	-	-	$a = 4.752$ $c = 4.429$	-

In CrN, the spin-up channel exhibits metallic behaviour, while the spin-down channel shows a partial gap, giving rise to half-metallicity. This spin-resolved asymmetry originates from exchange splitting of the Cr d-states. Orbital-resolved analysis reveals that the occupied states near the Fermi level primarily originate from the Cr t_{2g} orbitals (particularly d_{xz}, Figure S3b in Supplementary Information) while the e_g orbitals lie higher in energy and are largely unoccupied. Bader charge analysis reveals a charge transfer of 1.39e from each Cr atom in CrN_2, distributed across two neighbouring N atoms, indicating mixed ionic-covalent bonding. In CrN, a slightly higher transfer of 1.43e occurs to one N, reflecting more ionic character.

Table S2: Bader charges of Cr and N for pristine Cr_xN_y .

Composition	Cr charges (eV)	N charges (eV)
CrN_2	1.392	-0.628, -0.764
CrN	1.428	-1.428
Cr_2N	0.799, 0.800	-1.693, -1.551

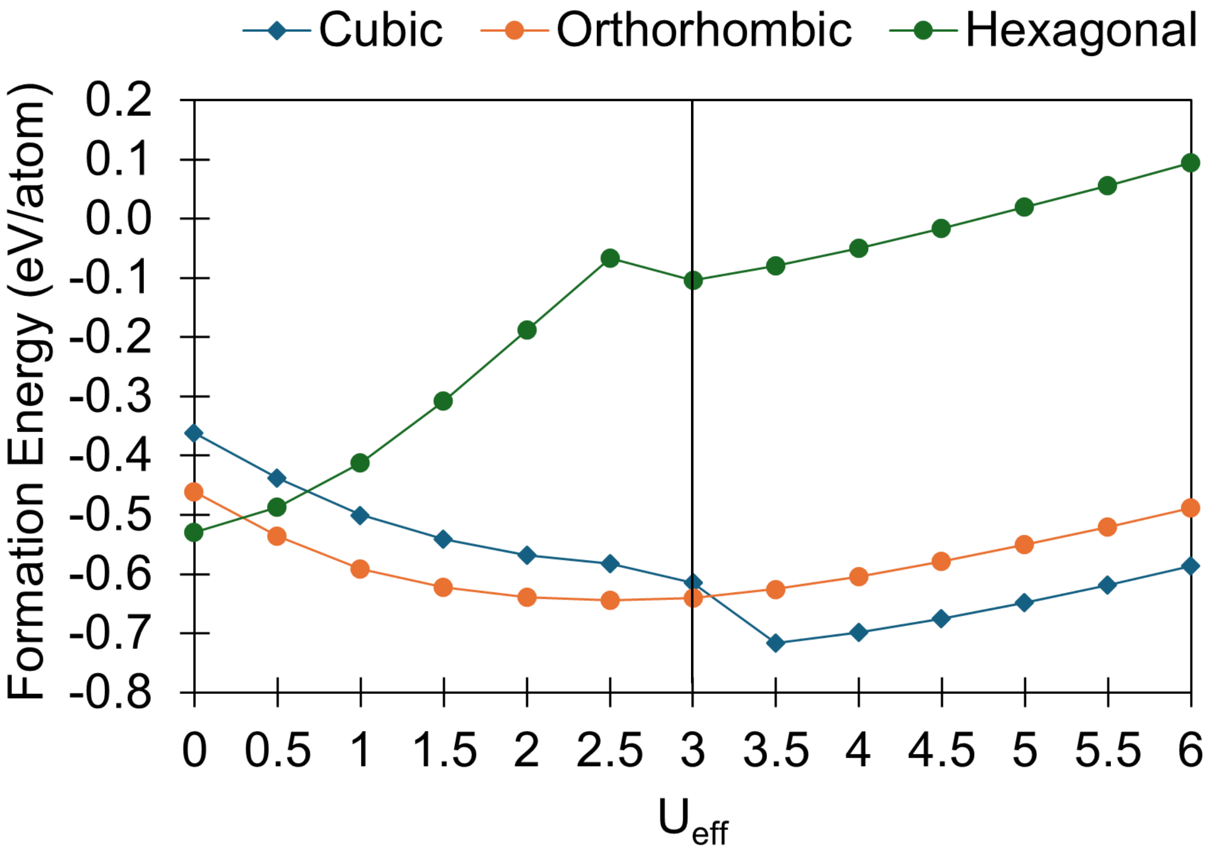

Figure S1: Formation energy vs U_{eff} of the three crystal structures of CrN (cubic and hexagonal are in FM ordering, orthorhombic is in the experimental AFM_{110} ordering).

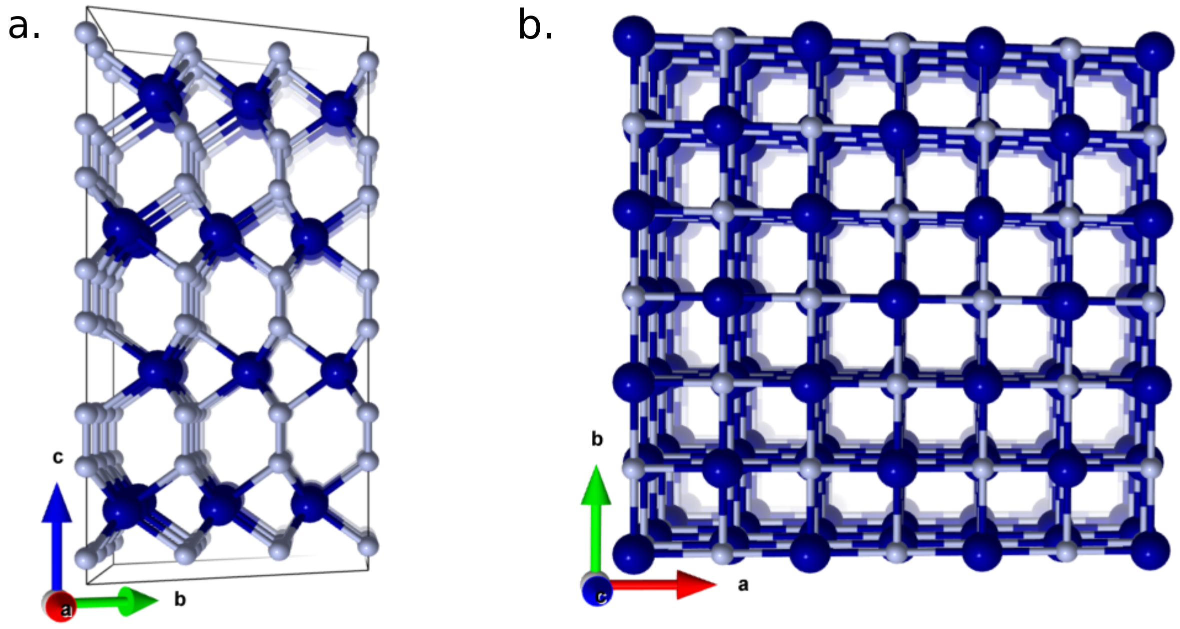

Figure S2: Full model of (a) CrN_2 and (b) CrN. Blue atoms are Cr and grey atoms are N.

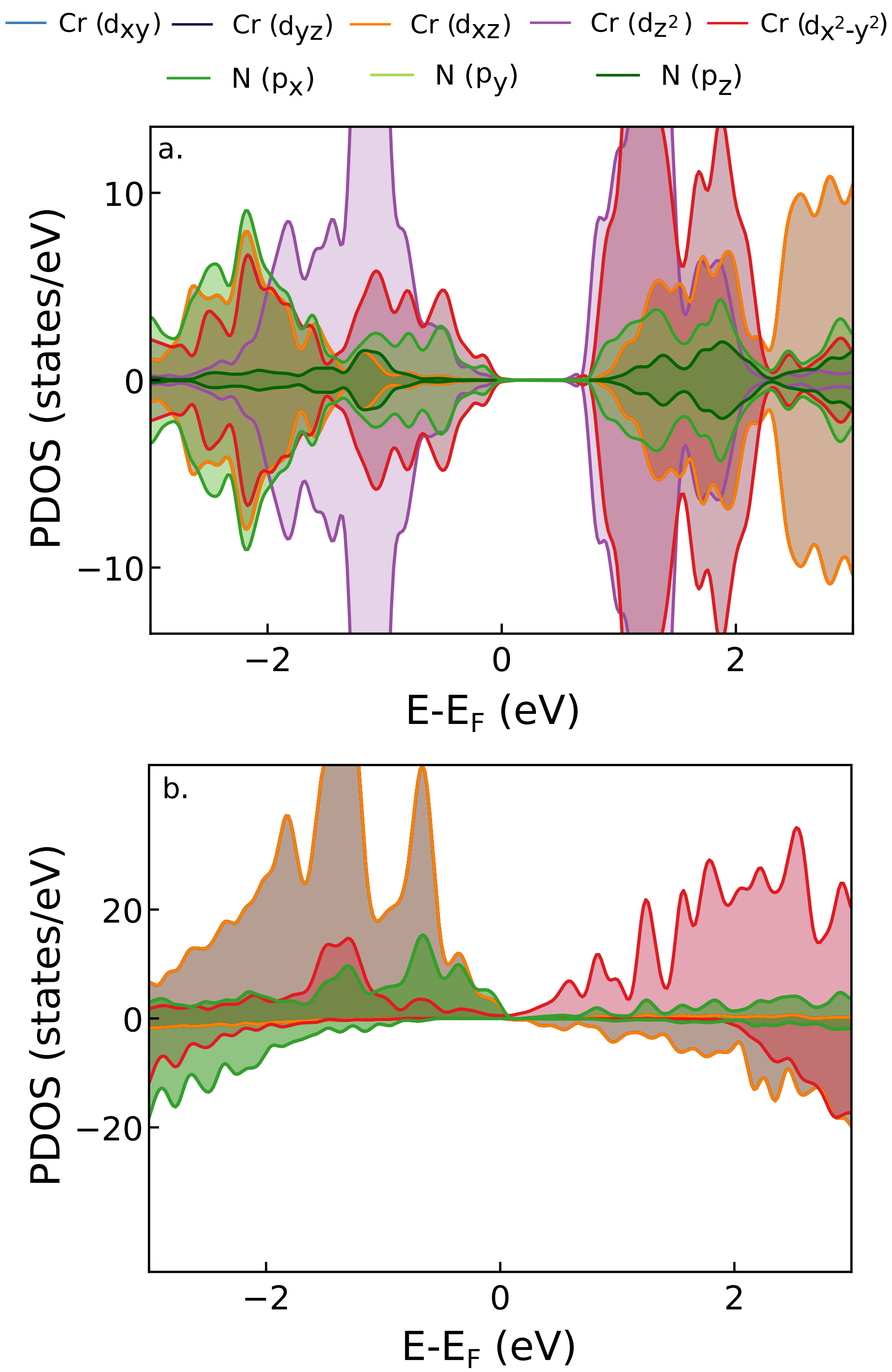

Figure S3: Projected density of states of lm-decomposed orbitals of pristine a. CrN_2 and b. CrN.

1.1 Elastic tensors of bulk CrN_2 and CrN(FM, AFM magnetic ordering)

CrN_2

$$C = \begin{bmatrix} 508.805 & 160.071 & 143.230 & 0.000 & 0.000 & 0.000 \\ 160.071 & 508.805 & 143.230 & 0.000 & 0.000 & 0.000 \\ 143.230 & 143.230 & 1126.368 & 0.000 & 0.000 & 0.000 \\ 0.059 & -0.059 & 0.000 & 244.998 & 0.000 & 0.000 \\ 0.000 & 0.000 & 0.000 & 0.000 & 244.998 & 0.059 \\ 0.000 & 0.000 & 0.000 & 0.000 & 0.059 & 174.367 \end{bmatrix}$$

CrN (FM)

$$C = \begin{bmatrix} 493.751 & 109.761 & 109.761 & 0.000 & 0.000 & 0.000 \\ 109.761 & 493.751 & 109.761 & 0.000 & 0.000 & 0.000 \\ 109.761 & 109.761 & 493.751 & 0.000 & 0.000 & 0.000 \\ 0.000 & 0.000 & 0.000 & 156.217 & 0.000 & 0.000 \\ 0.000 & 0.000 & 0.000 & 0.000 & 156.217 & 0.000 \\ 0.000 & 0.000 & 0.000 & 0.000 & 0.000 & 156.217 \end{bmatrix}$$

CrN (Cubic - AFM_{110})

$$C = \begin{bmatrix} 542.025 & 78.044 & 92.406 & 0.000 & 0.000 & 0.000 \\ 78.044 & 542.025 & 92.406 & 0.000 & 0.000 & 0.000 \\ 92.406 & 92.406 & 532.353 & 0.000 & 0.000 & 0.000 \\ 0.000 & 0.000 & 0.000 & 145.333 & 0.000 & 0.000 \\ 0.000 & 0.000 & 0.000 & 0.000 & 145.333 & 0.000 \\ 0.000 & 0.000 & 0.000 & 0.000 & 0.000 & 140.519 \end{bmatrix}$$

2 Defects Characterisation

To ensure convergence and assess the impact of magnetic ordering on defect energetics, we computed the nitrogen vacancy formation energy across different supercell sizes for both CrN_2 and CrN, with results summarised in Table S3. For CrN, we also compared FM, AFM, and paramagnetic-like (SQS+DLM) ordering. The relative stability of the vacancy is preserved. These results justify the use of FM CrN in the main manuscript as an efficient approximation of the room-temperature paramagnetic state.

Table S3: Nitrogen vacancy formation energies (eV) of CrN_2 and CrN in various supercell sizes and N-rich chemical potential.

Supercell Size	Magnetic Ordering	$E_f^{def}(V_N)$ (eV)
**CrN_2**		
2x2x2 (48 atoms)	NM	2.17
3x3x1 (54 atoms)	NM	2.32
3x3x2 (108 atoms)	NM	2.34
3x3x3 (162 atoms)	NM	2.35
**CrN**		
2x2x2 (64 atoms)	FM	1.97
3x3x3 (216 atoms)	FM	1.71
4x4x4 (512 atoms)	FM	1.71
2x2x2 (64 atoms)	AFM	1.94
4x4x2 (256 atoms)	AFM	1.65
2x2x2 (64 atoms)	PM (SQS+DLM)	1.91

After converging for size effects considering different supercells, calculations employed the 3×3×3 (216 atoms) and 3×3×2 (108 atoms) supercells for CrN and CrN_2, respectively, as illustrated in Figure S3. For clarity, the visualizations of other defect structures are cropped to highlight only the local environment around the defect site.

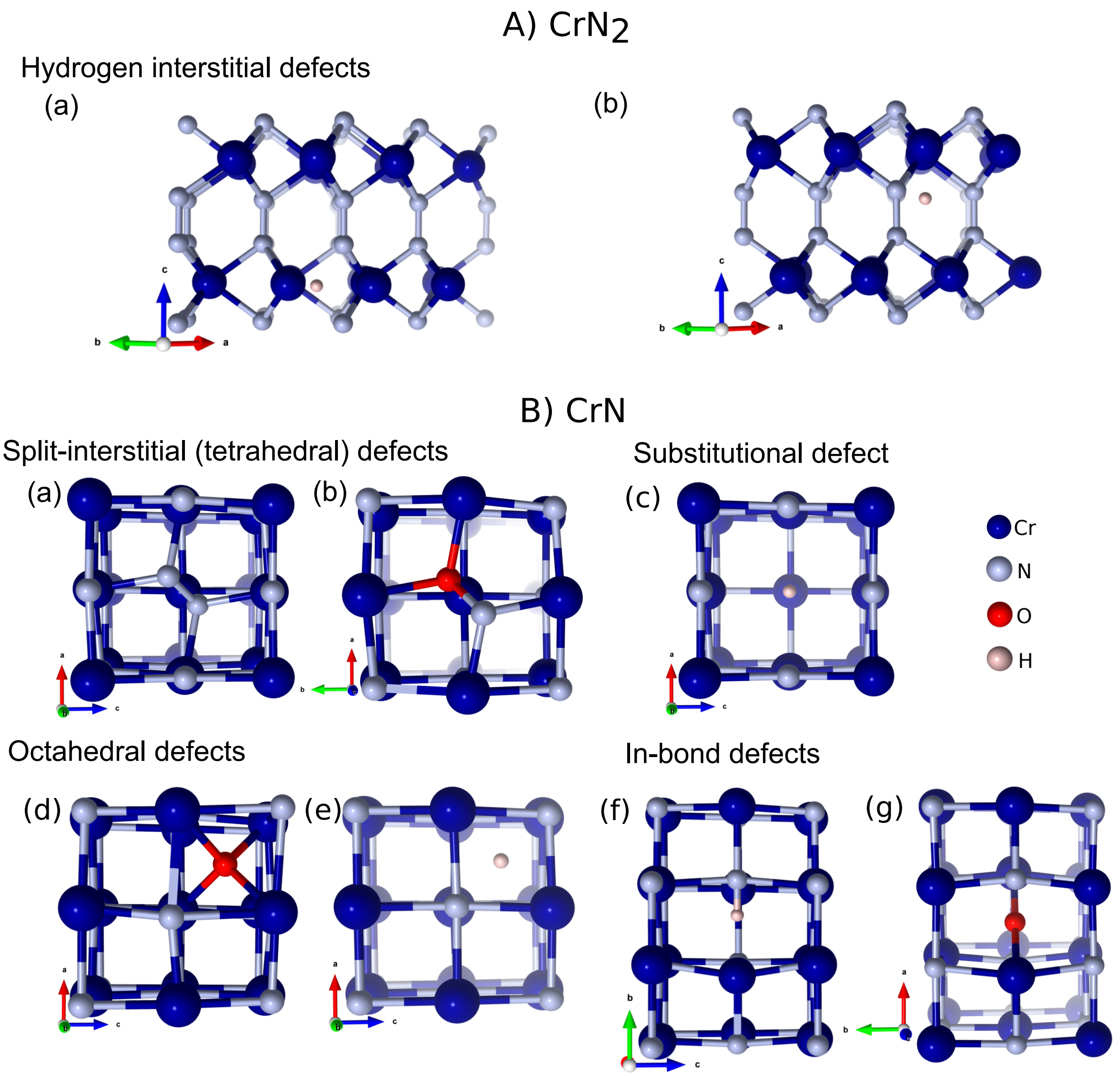

Figure S4: Higher defect formation energy defect structures (with energy less than 3 eV, see Table S4) of CrN_2 and CrN. The images show cutouts of the supercells to clearly highlight the defects (not the full cells used for the simulations).

Table S4: Defect formation energies were calculated according to equation 2 in the main manuscript.

Composition Defect type	Defect	N-poor	Stoichiometric	N-rich
**CrN_2**				
Chromium	$V_{Cr}^{\times}$	6.10	5.58	5.06
	$Cr_N^{\times}$	5.40	6.18	6.96
	$Cr_i^{\times}$	6.41	6.93	7.45
Nitrogen	$V_N^{\times}$	1.82	2.08	2.34
	$N_{Cr}^{\times}$	10.96	10.17	10.43
	$N_i^{\times}$	8.39	8.13	7.87
	$N_i^{\times}$	8.86	8.60	8.34
Hydrogen	$H_N^{\times}$	0.30	1.24	1.50
	$H_{Cr}^{\times}$	8.02	7.50	6.98
	$H_i^{\times}$	2.58	2.58	2.58
	$H_i^{\times}$	2.92	2.92	2.92
Oxygen	$O_N^{\times}$	-0.46	-0.20	0.06
	$O_{Cr}^{\times}$	8.99	8.47	7.95
	$O_i^{\times}$	5.42	5.42	5.42
	$O_i^{\times}$	5.27	5.27	5.27
**CrN**				
Chromium	$V_{Cr}^{\times}$	3.30	2.71	2.13
	$Cr_N^{\times}$	6.31	7.48	8.65
	$Cr_i^{\times}$	3.99	4.58	5.16
Nitrogen	$V_N^{\times}$	0.54	1.12	1.71
	$N_{Cr}^{\times}$	10.19	9.03	7.86
	$N_i^{\times}$	4.58	3.99	3.41
Hydrogen	$H_i^{\times}$	1.17	1.17	1.17
	$H_i^{\times}$	1.38	1.38	1.38
	$H_i^{\times}$	2.32	2.32	2.32
	$H_N^{\times}$	0.19	0.77	1.36
	$H_{Cr}^{\times}$	3.88	4.46	5.05
Oxygen	$O_i^{\times}$	1.35	1.35	1.35
	$O_i^{\times}$	3.47	3.47	3.47
	$O_i^{\times}$	3.91	3.91	3.91
	$O_N^{\times}$	-3.39	-3.98	-2.81
	$O_{Cr}^{\times}$	5.84	6.43	7.01

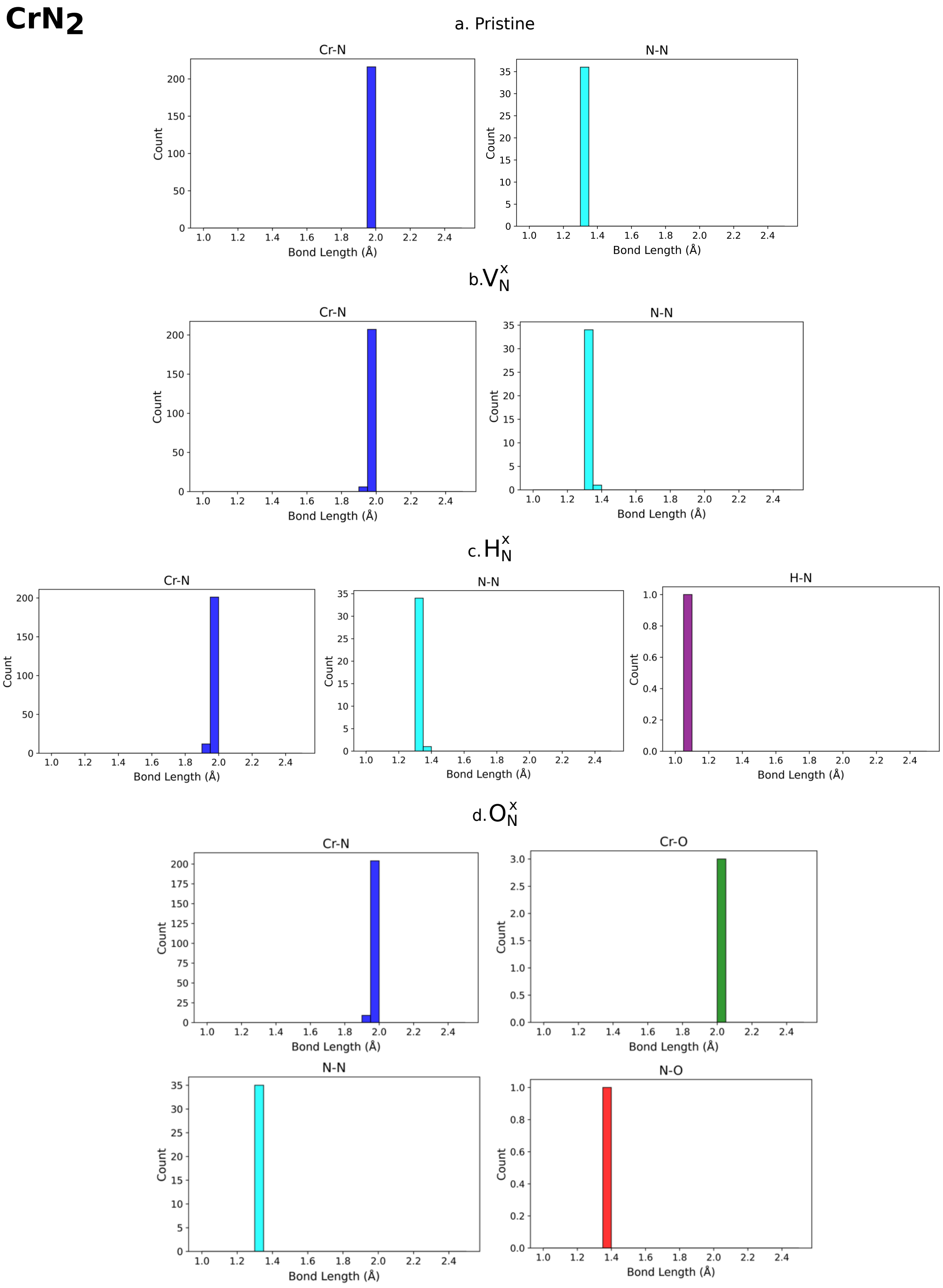

Figure S5: Distribution of bond lengths in pristine CrN_2 and in presence of the defects as presented in Table 1 in the main manuscript.

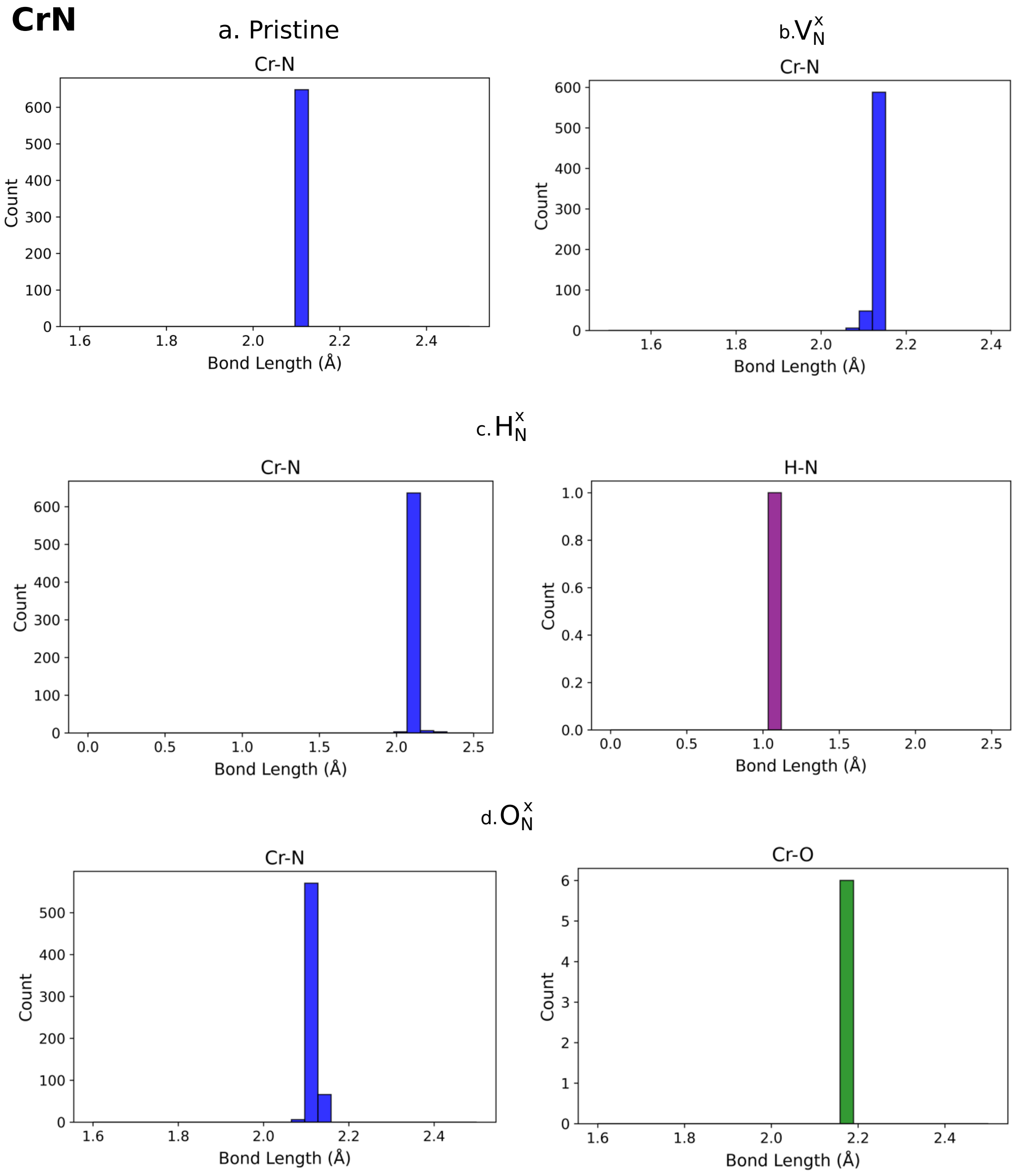

Figure S6: Distribution of bond lengths in pristine CrN and in presence of the defects as presented in Table 1 in the main manuscript.

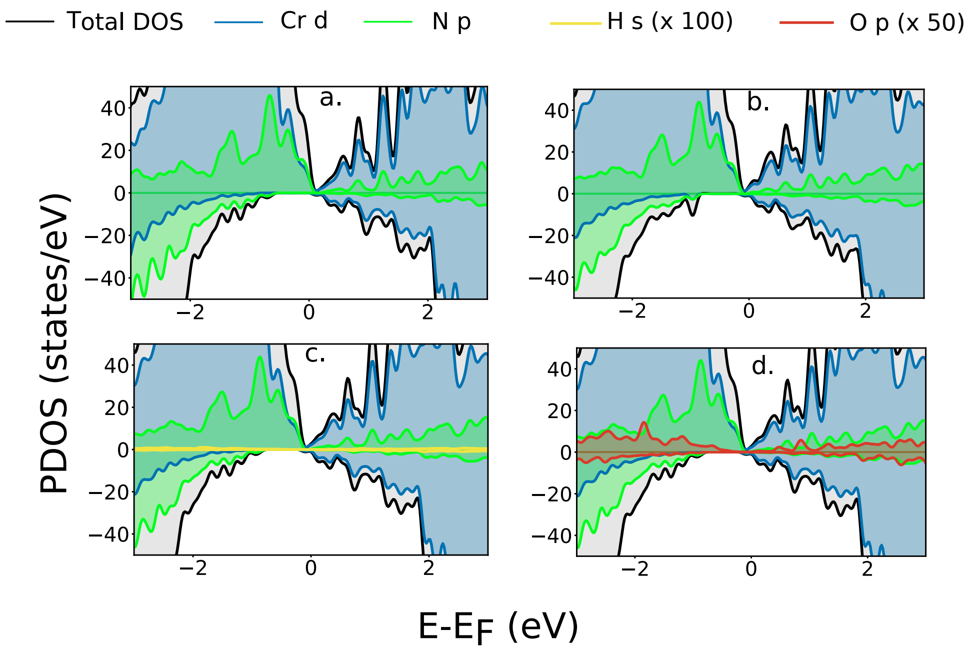

Figure S7: Projected density of state plots of (a) pristine (b) V_N (c) H_i (d) O_N in CrN.

Table S5: Bader charges (q_i, i=Cr, N, O, H) for the pristine and defective compositions of Cr_xN_y. q_i for the defective structures refers the range of net charge in the entire system for the lowest energy neutral defects.

System	q_{Cr} (e)	q_N (e)	$q_{O,H}$ (e)
**CrN_2**			
Pristine	+1.39	-0.63 - -0.76	
$V_N^{\times}$	+1.30 - +1.42	-0.63 - -0.80	
$H_N^{\times}$	+1.25 - +1.42	-0.63 - -0.83	0.24
$O_N^{\times}$	+1.39 - +1.40	-0.53 - -0.77	-0.79
**CrN**			
Pristine	+1.43	-1.43	
$V_N^{\times}$	+1.26 - +1.44	-1.43 - -1.46	
$H_i^{\times}$	+1.42 - +1.54	-1.43 - -1.50	0.27
$O_N^{\times}$	+1.42 - +1.50	-1.43 - -1.45	-1.39

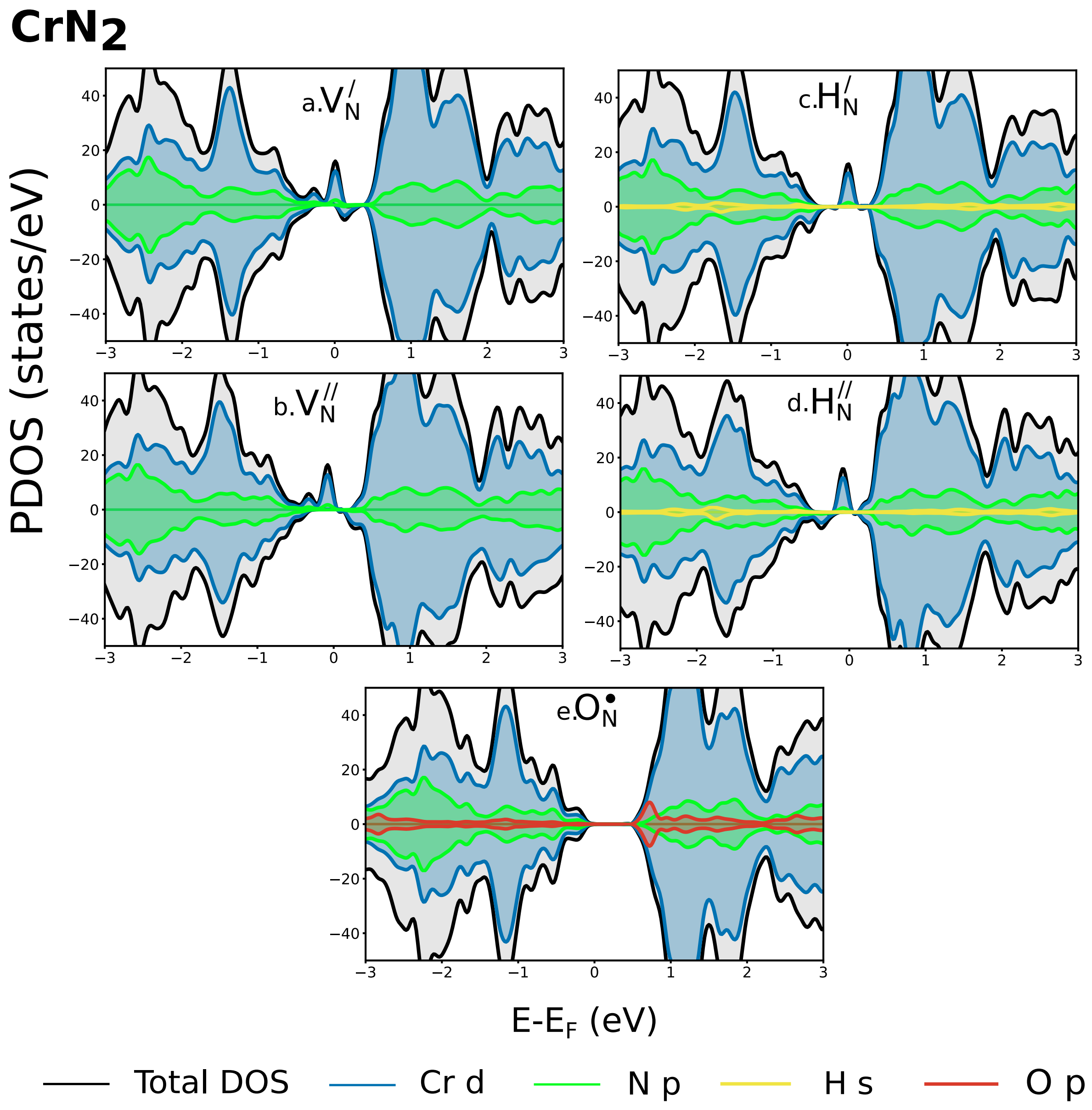

Figure S8: PDOS of the charged defects in CrN_2. The corresponding CTL plot is presented as Figure 4 in the main manuscript.

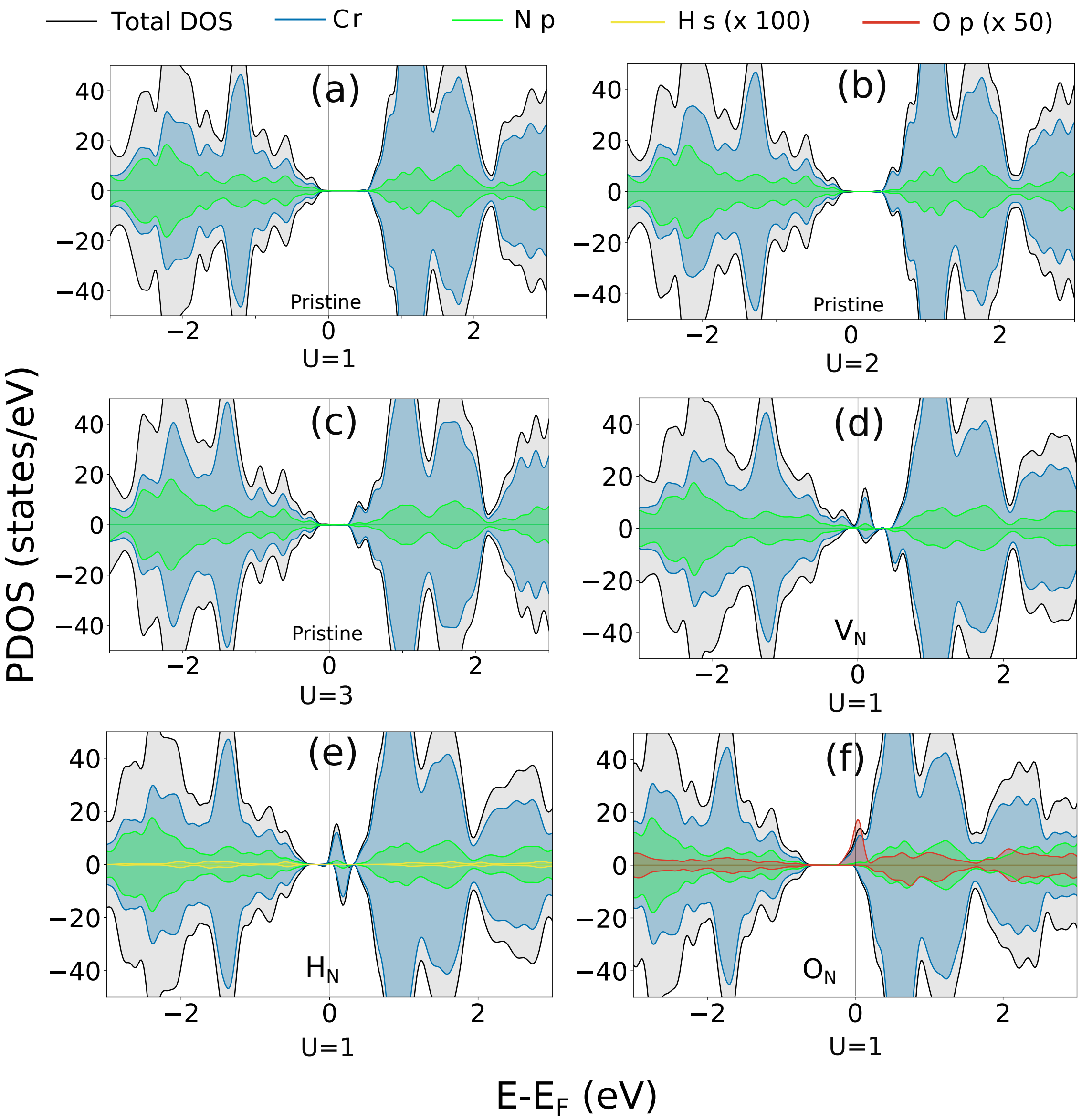

Figure S9: Projected density of state plots of pristine CrN_2 in presence of Hubbard U correction.

2.1 Elastic tensors of the lowest energy defects in CrN_2 and CrN

CrN_2

$V_{\mathrm{N}}^{\times}$

$$C = \begin{bmatrix} 496.796 & 152.821 & 140.228 & 0.059 & 0.000 & 0.000 \\ 152.821 & 496.796 & 140.228 & -0.059 & 0.000 & 0.000 \\ 140.228 & 140.228 & 1044.126 & 0.000 & 0.000 & 0.000 \\ 0.059 & -0.059 & 0.000 & 224.788 & 0.000 & 0.000 \\ 0.000 & 0.000 & 0.000 & 0.000 & 224.788 & 0.059 \\ 0.000 & 0.000 & 0.000 & 0.000 & 0.059 & 171.987 \end{bmatrix}$$

$\mathrm{H}_{\mathrm{N}}^{\times}$

$$C = \begin{bmatrix} 498.125 & 153.976 & 145.385 & 0.588 & 0.000 & 0.000 \\ 153.976 & 498.125 & 145.385 & -0.588 & 0.000 & 0.000 \\ 145.385 & 145.385 & 1076.761 & 0.000 & 0.000 & 0.000 \\ 0.588 & -0.588 & 0.000 & 232.995 & 0.000 & 0.000 \\ 0.000 & 0.000 & 0.000 & 0.000 & 232.995 & 0.588 \\ 0.000 & 0.000 & 0.000 & 0.000 & 0.588 & 172.074 \end{bmatrix}$$

$\mathrm{O}_{\mathrm{N}}^{\times}$

$$C = \begin{bmatrix} 496.826 & 161.843 & 154.695 & 3.298 & 0.000 & 0.000 \\ 161.843 & 496.826 & 154.695 & -3.298 & 0.000 & 0.000 \\ 154.695 & 154.695 & 1091.115 & 0.000 & 0.000 & 0.000 \\ 0.588 & -0.588 & 0.000 & 237.088 & 0.000 & 0.000 \\ 0.000 & 0.000 & 0.000 & 0.000 & 237.088 & 3.298 \\ 0.000 & 0.000 & 0.000 & 0.000 & 3.298 & 167.491 \end{bmatrix}$$

$\mathrm{O}_{\mathrm{N}}^{\bullet}$

$$C = \begin{bmatrix} 499.850 & 156.096 & 143.746 & 0.607 & 0.000 & 0.000 \\ 156.096 & 499.850 & 143.746 & -0.607 & 0.000 & 0.000 \\ 143.746 & 143.746 & 1111.605 & 0.000 & 0.000 & 0.000 \\ 0.607 & -0.607 & 0.000 & 240.352 & 0.000 & 0.000 \\ 0.000 & 0.000 & 0.000 & 0.000 & 240.352 & 0.607 \\ 0.000 & 0.000 & 0.000 & 0.000 & 0.607 & 171.877 \end{bmatrix}$$

CrN

$V_{\mathrm{N}}^{\times}$

$$C = \begin{bmatrix} 487.801 & 107.048 & 107.048 & 0.000 & 0.000 & 0.000 \\ 107.048 & 487.801 & 107.048 & 0.000 & 0.000 & 0.000 \\ 107.048 & 107.048 & 487.801 & 0.000 & 0.000 & 0.000 \\ 0.000 & 0.000 & 0.000 & 155.547 & 0.000 & 0.000 \\ 0.000 & 0.000 & 0.000 & 0.000 & 155.547 & 0.000 \\ 0.000 & 0.000 & 0.000 & 0.000 & 0.000 & 155.547 \end{bmatrix}$$

$H_i^{\times}$

$$C = \begin{bmatrix} 454.520 & 142.583 & 113.762 & 0.258 & 0.000 & 0.000 \\ 142.583 & 454.520 & 113.762 & -0.258 & 0.000 & 0.000 \\ 113.762 & 113.762 & 473.150 & 0.000 & 0.000 & 0.000 \\ 0.258 & -0.258 & 0.000 & 156.633 & 0.000 & 0.000 \\ 0.000 & 0.000 & 0.000 & 0.000 & 156.633 & 0.258 \\ 0.000 & 0.000 & 0.000 & 0.000 & 0.258 & 155.968 \end{bmatrix}$$

$O_N^{\times}$

$$C = \begin{bmatrix} 492.565 & 107.947 & 107.947 & 0.000 & 0.000 & 0.000 \\ 107.947 & 492.565 & 107.947 & 0.000 & 0.000 & 0.000 \\ 107.947 & 107.947 & 492.565 & 0.000 & 0.000 & 0.000 \\ 0.000 & 0.000 & 0.000 & 156.633 & 0.000 & 0.000 \\ 0.000 & 0.000 & 0.000 & 0.000 & 156.633 & 0.000 \\ 0.000 & 0.000 & 0.000 & 0.000 & 0.000 & 156.633 \end{bmatrix}$$

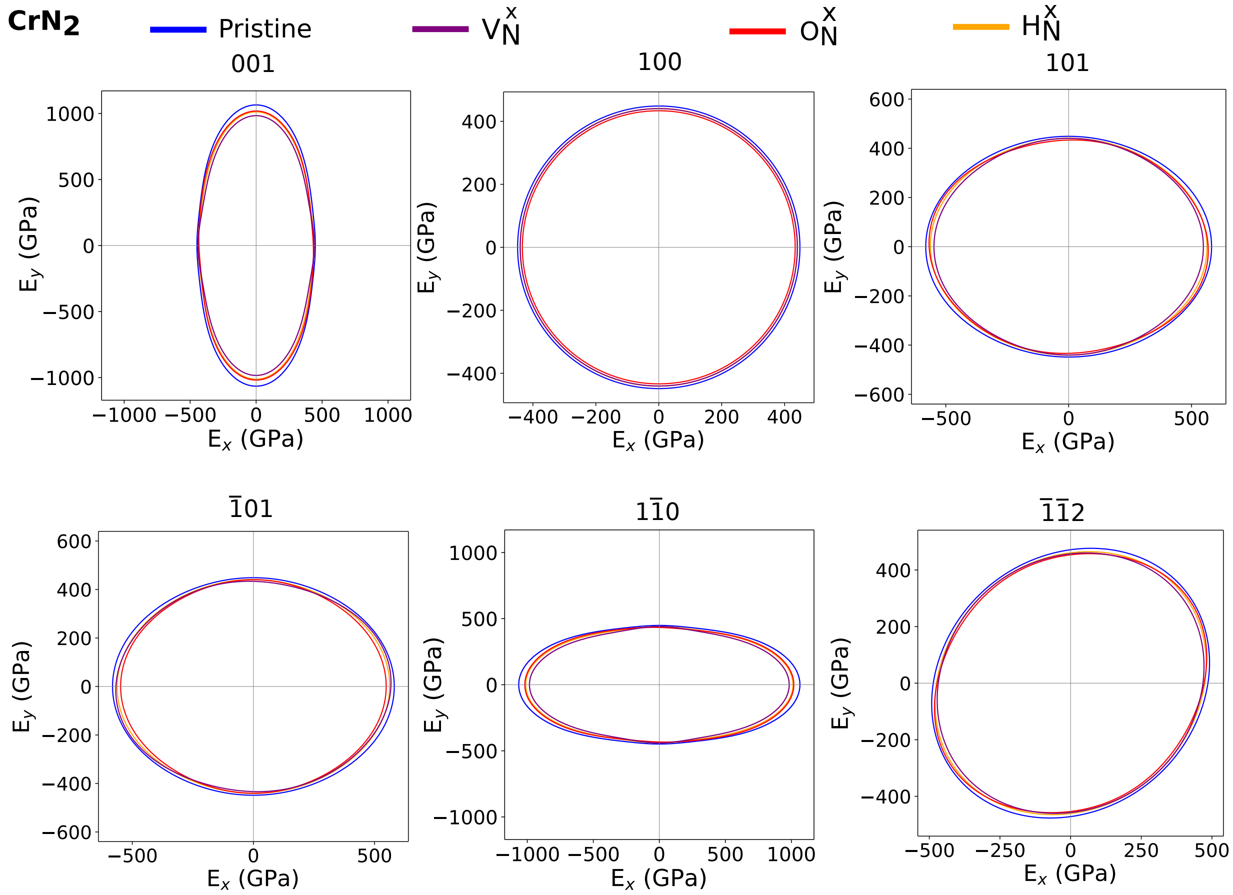

Figure S10: Direction elastic moduli plots for CrN_2.

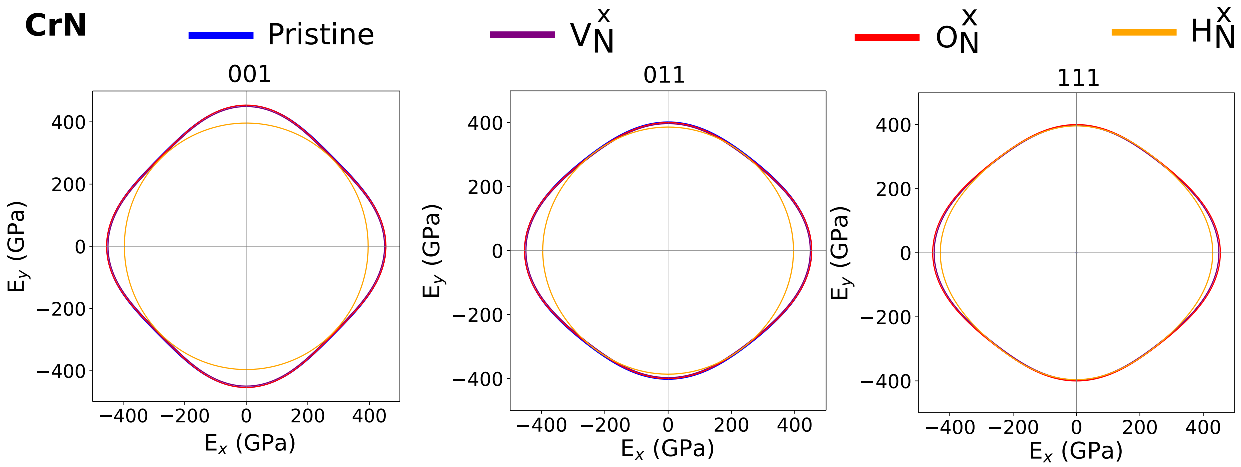

Figure S11: Direction elastic moduli plots for CrN.

References

[1] L. M. Corliss, N. Elliott and J. M. Hastings, *Physical Review*, 1960, **117**, 929–935.

[2] L. Zhou, F. Körmann, D. Holec, M. Bartosik, B. Grabowski, J. Neugebauer and P. H. Mayrhofer, *Physical Review B*, 2014, **90**, 184102.

[3] F. Rivadulla, M. Bãobre-López, C. X. Quintela, A. Pĩeiro, V. Pardo, D. Baldomir, M. A. López-Quintela, J. Rivas, C. A. Ramos, H. Salva, J. S. Zhou and J. B. Goodenough, *Nature Materials 2009 8:12*, 2009, **8**, 947–951.

[4] X. Y. Zhang, J. S. Chawla, R. P. Deng and D. Gall, *Physical Review B - Condensed Matter and Materials Physics*, 2011, **84**, 073101.

[5] X. F. Duan, W. B. Mi, Z. B. Guo and H. L. Bai, *Journal of Applied Physics*, 2013, **113**, 023701.

[6] B. Biswas, S. Chakraborty, A. Joseph, S. Acharya, A. I. K. Pillai, C. Narayana, V. Bhatia, M. Garbrecht and B. Saha, *Acta Materialia*, 2022, **227**, 117737.

[7] S. Wang, X. Yu, J. Zhang, L. Wang, K. Leinenweber, D. He and Y. Zhao, *Crystal Growth & Design*, 2016, **16**, 351–358.

[8] A. Herwadkar and W. R. Lambrecht, *Physical Review B - Condensed Matter and Materials Physics*, 2009, **79**, 035125.

[9] K. Niwa, T. Yamamoto, T. Sasaki and M. Hasegawa, *Physical Review Materials*, 2019, **3**, 053601.

[10] K. Alam, R. Ponce-Pérez, K. Sun, A. Foley, N. Takeuchi and A. R. Smith, *Journal of Vacuum Science & Technology A*, 2023, **41**,.

[11] S. J. Kim, T. Marquart and H. F. Franzen, *Journal of the Less Common Metals*, 1990, **158**, L9–L10.

[12] M. A. Gharavi, G. Greczynski, F. Eriksson, J. Lu, B. Balke, D. Fournier, A. le Febvrier, C. Pallier and P. Eklund, *Journal of Materials Science*, 2019, **54**, 1434–1442.

[13] T. H. Lee, S. J. Kim, E. Shin and S. Takaki, *Acta crystallographica. Section B, Structural science*, 2006, **62**, 979–986.
