# Supplementary material for: Elucidating the impact of point defects on the structural, electronic, and mechanical behaviour of chromium nitride
Source: Phys Chem Chem Phys. 2025 Oct 7;27(42):22610–20. doi: 10.1039/d5cp02904j (PMC12517257; doi:10.1039/d5cp02904j)

Supplementary Information (SI) for Physical Chemistry Chemical Physics.
This journal is © the Owner Societies 2025

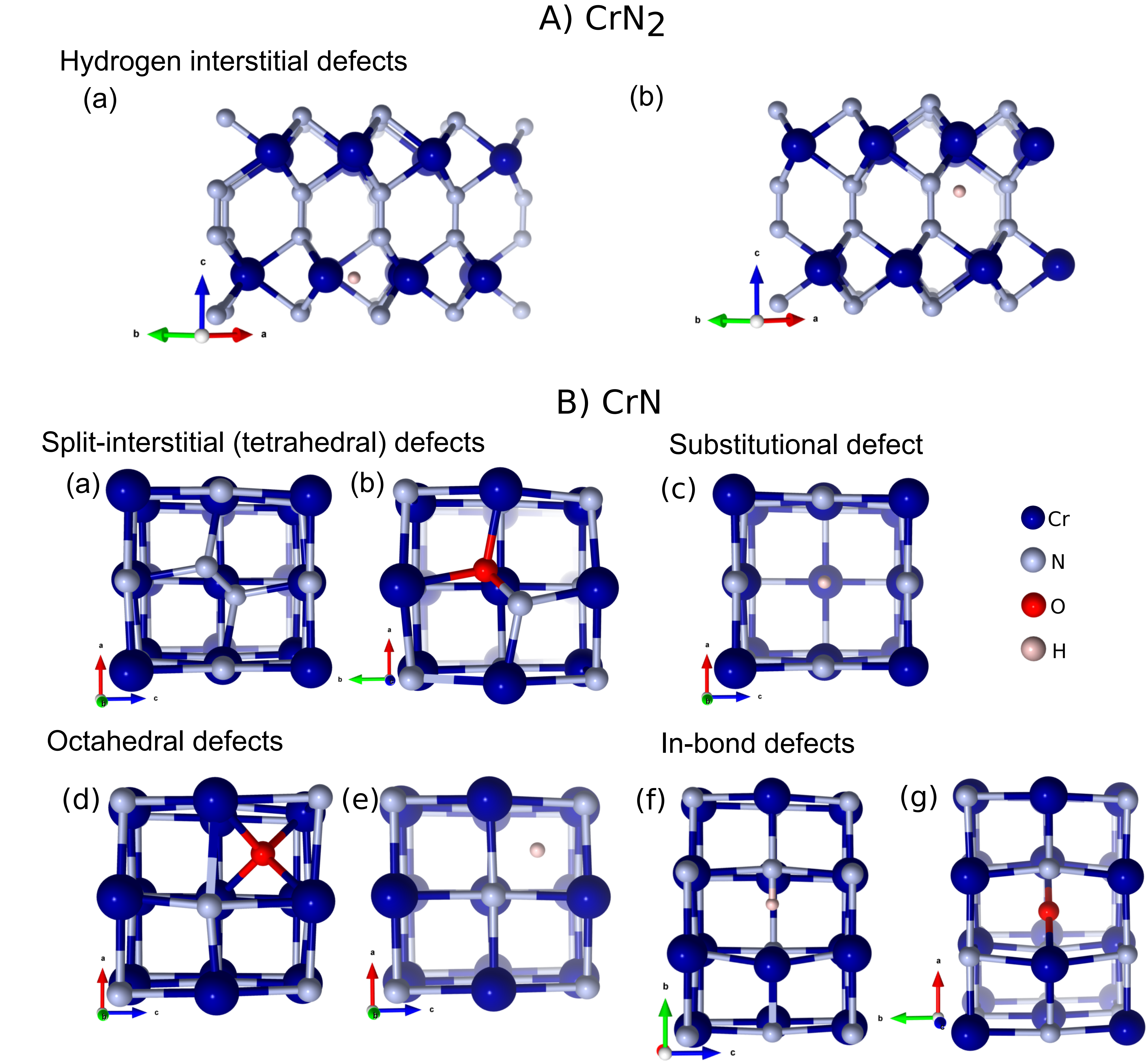

Supplement: CP-027-D5CP02904J-s009 [file CP-027-D5CP02904J-s009.pdf]
